# Amelioration of AOM/DSS-Induced Murine Colitis-Associated Cancer by Evodiamine Intervention is Primarily Associated with Gut Microbiota-Metabolism-Inflammatory Signaling Axis

**DOI:** 10.3389/fphar.2021.797605

**Published:** 2021-12-24

**Authors:** Mengxia Wang, Biqiang Zhou, Weihong Cong, Miao Zhang, Ziwen Li, Yan Li, Shaoyu Liang, Keji Chen, Depo Yang, Zhengzhi Wu

**Affiliations:** ^1^ School of Pharmaceutical Sciences, Sun Yat-sen University, Guangzhou, China; ^2^ The First Affiliated Hospital of Shenzhen University, Shenzhen Second People’s Hospital, Shenzhen, China; ^3^ Shenzhen Institute of Geriatrics, Shenzhen, China; ^4^ National Clinical Research Center for Chinese Medicine Cardiology, Xiyuan Hospital, China Academy of Chinese Medical Sciences, Beijing, China

**Keywords:** colitis-associated cancer, Evodiamine, gut microbiota, tryptophan metabolism, inflammatory signaling pathway

## Abstract

Evodiamine (EVO), an indole alkaloid derived from Rutaceae plants *Evodia rutaecarpa* (Juss.) Benth.、*Evodia rutaecarpa* (Juss.) Benth. Var. bodinieri (Dode) Huang or *Evodia rutaecarpa* (Juss.) Benth. Var. officinalis (Dode) Huang, has anti-inflammatory and anti-tumor activities. Our previous study found that EVO attenuates colitis by regulating gut microbiota and metabolites. However, little is known about its effect on colitis-associated cancer (CAC). In this study, the protective effects of EVO on azoxymethane (AOM)/dextran sulfate sodium (DSS)-induced colitis and tumor mice were observed, and the underlying potential mechanism was clarified. The results suggested that EVO ameliorated AOM/DSS-induced colitis by inhibiting the intestinal inflammation and improving mucosal barrier function. And EVO significantly reduced the number and size of AOM/DSS-induced colorectal tumors along with promoted apoptosis and inhibited proliferation of epithelial cell. Moreover, EVO promoted the enrichment of SCFAs-producing bacteria and reduced the levels of the pro-inflammatory bacteria, which contributes to the changes of microbiota metabolism, especially tryptophan metabolism. Furthermore, inflammatory response (like Wnt signaling pathway、Hippo signaling pathway and IL-17 signaling pathway) were effectively alleviated by EVO. Our study demonstrated that the protective therapeutic action of EVO on CAC is to inhibit the development of intestinal inflammation-cancer by regulating gut microbiota metabolites and signaling pathways of colon intestinal epithelial, which may represent a novel agent for colon cancer prevention via manipulation of gut microbiota.

## Introduction

Colorectal cancer (CRC) is the third most commonly diagnosed cancer worldwide, with high morbidity and mortality rates (Siegel et al., 2020). Studies have found that CRC may be affected by environmental factors (e.g., diet, smoking, alcohol), heredity (e.g. genetic mutations, microRNAs expression) and immune system (Chen et al., 2017). Accumulating evidences indicate that chronic inflammation of the colon may have a closely linked to the pathogenesis of CRC. Inflammatory bowel diseases (IBD), including ulcerative colitis (UC) and Crohn’s disease (CD), which is associated with an increased risk of CRC, may progress to colitis-associated colorectal cancer (CAC) (Rieder et al., 2017). While CAC is known to be related to inflammation, the specific mechanism of inflammation involved in the pathogenesis of CAC have not been clarified.

In recent years, new lines of evidence have shown that gut microbiota play a key role in the development of IBD and CRC (Drewes et al., 2016; Li et al., 2020). Studies have also shown that changes in microbial composition and diversity are associated with the progression of CAC. An analysis of five different IBD patient cohorts form five different countries revealed that there are major differences in the diversity and abundance of the gut microbiota between healthy individuals and IBD patients (Sankarasubramanian et al., 2020). Interestingly, similar to IBD patients, the microbiota composition of CRC patients had also similar changes, characterized by increased abundance of Proteobacteria and decreased Firmicutes (Gagniere et al., 2016). The adherent/invasive strains of *Escherichia coli* were highly abundant in the colonic mucosa of patients with colorectal carcinoma and adenoma (Wang Z. et al., 2019). Recently, an interesting study showed that a purified membrane protein derived from the probiotics *Akkermansia muciniphila* could blunt the occurrence of colitis associated tumors (Wang L. et al., 2020). A longitudinal study of a CAC mouse model has also showed a significant change in microbiota composition in the presence of chemically induced chronic intestinal inflammation (Liang et al., 2014). These findings suggest that the gut microbiota may serve as a pivotal mediator for the progression of intestinal diseases and regulate the composition of gut microbiota has emerged in the prevention of CAC.

CAC is a progressive process from inflammation to cancer, so the prevention of CAC is of vital importance. At present, one of the most prevalent treatments for IBD in clinical is administration of some agents, including 5-aminosalicylic acid, corticosteroids, biological agents and immunosuppressants (Gao et al., 2020). However, treatment with these drugs tends to increase the patient’s susceptibility to infection and leads to different adverse drug reactions (Gong et al., 2018). Once a malignant tumor is detected, total colectomy should be considered, but prophylactic surgery (proctocolectomy) does not always eliminate the risk of carcinogenesis (Sokol et al., 2014; Wang X. et al., 2019). Due to the limited efficacy and side effects of these therapies, alternative drugs that have the potential to treat both early inflammatory and late oncogenic stages should be developed; at the same time, the development from IBD to CRC is a long process, which provides an opportunity for intervention to inhibit the development of CRC.

Evodiamine (EVO), a quinolone alkaloid from the traditional botanical drug *Evodia rutaecarpa* (Juss.) Benth., *Evodia rutaecarpa* (Juss.) Benth. Var. bodinieri (Dode) Huang or *Evodia rutaecarpa* (Juss.) Benth. Var. officinalis (Dode) Huang (Chinese name: Wu-Zhu-Yu; Rutaceae), has been reported to have beneficial pharmacological properties, such as anti-obesity, anti-inflammation, anti-thrombotic and anti-cancer (Yu et al., 2016). It has been reported that the EVO inhibited high-fat diet-induced colitis-associated cancer in mice through regulating the gut microbiota (Zhu et al., 2020). And we have previously demonstrated that EVO can be effectively used to prevent and treat UC rats by regulating the gut microbiota and metabolism (Wang L. et al., 2020). Moreover, EVO could induce apoptosis and inhibit migration of colorectal cancer cells (Zhao et al., 2015). However, whether EVO inhibits the occurrence of colon cancer by inhibiting intestinal inflammation is still unclear, and the underlying mechanism has not yet been elucidated. The complex interaction between gut microbiota and anti-inflammatory and anti-tumor activities during CAC are also not fully understood.

Therefore, in this study with a mouse azomethanze (AOM)/sodium dextran sulfate (DSS)-induced colitis model and AOM/DSS-induced tumor model, we demonstrated that EVO treatment significantly protected against colitis, colon carcinogenesis and tumor growth. We want to know whether EVO affects the gut microbiota of CAC mice. Moreover, we also want to investigate if EVO changes metabolic activities and pathways after regulating the gut microbiota composition. On the other hand, we want to understand whether EVO impacts the gene-gene interaction of colonic epithelial cells after changing the composition of gut microbiota. This study can be considered as a reference for EVO to further study the mechanism of CAC, which is a promising agent for the prevention and treatment of CAC.

## Materials and Methods

### Mice and AOM/DSS-Induced Colitis-Associated Colorectal Cancer

Specific pathogen-free (SPF) male C57BL/6 mice (6–8 weeks old, obtained from the Center of Laboratory Animal Science of Guangdong) were maintained in a specific sterile colony under a complete controlled condition (temperature 23 ± 2°C; humidity 50–70%; 12 h-light dark cycle and lighting at 8:00 am) and were allowed access to a commercial diet and water ad libitum.

**FIGURE 1:**
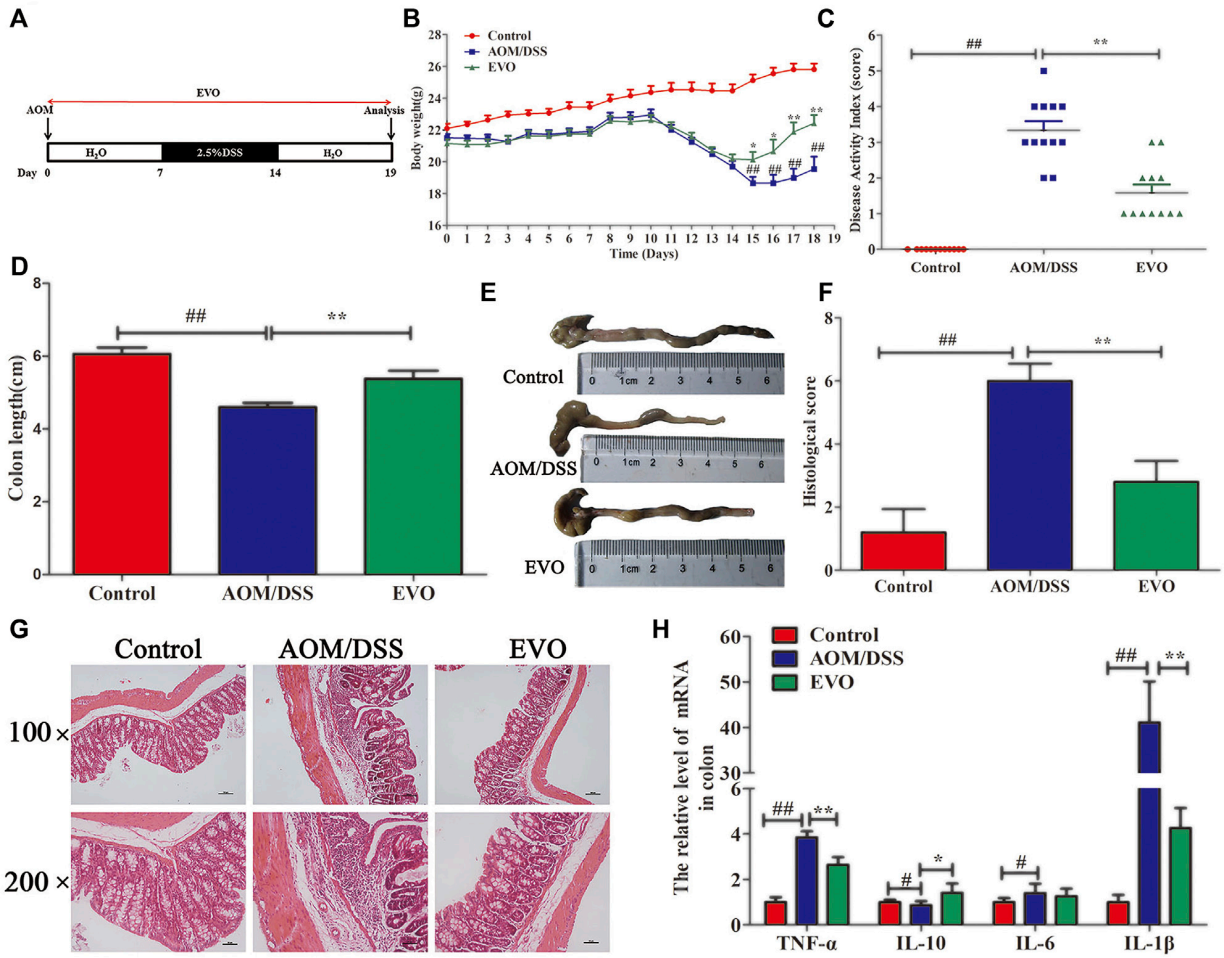
EVO alleviates colitis at the early stage of CAC. **(A)** Experimental protocol for model. **(B)** Body weight change. **(C)** Disease activity index score. **(D–E)** Colon length. **(F)** Histological scores. **(G)** H&E staining. **(H)** Inflammatory cytokines in colon. The results are presented as mean ± SD. ^#^ *p <* 0.05, ^##^ *p <* 0.01, Control *vs*. AOM/DSS; ^*^ *p <* 0.05, ^**^ *p <* 0.01, EVO *vs*. AOM/DSS.

For the early enteritis model, after a seven-day adaptation period, mice were randomly allocated to three groups (*n* = 12): Control group, AOM/DSS group and EVO group. AOM/DSS and EVO groups mice were intraperitoneally (i.p.) injected with 10 mg/ kg AOM (Sigma-Aldrich) at the start point of Week 1 and then received 2.5%DSS (MP Biomedical, 36,000–50000 MW) for 7 days. From the first day of the model induction, the mice of EVO group were gavaged with 40 mg/ kg Evodiamine (Meilunbio, MB5729), Control group and AOM/DSS group received orally 0.5%CMC-Na respectively daily for 18 days (Figure 1A), the dose of EVO was determined by the previous dose-effect relationship study in our research group (Wang MX. et al., 2020). Body weights were monitored daily and disease activity index (including weight loss, intestinal bleeding and stool consistency) (Supplementary Table S1) (Cooper et al., 1993). Feces samples were collected on 18^th^ days and stored at -80°C for further analysis.

For advanced tumor model, after a seven-day adaptation period, mice were randomly allocated to three groups (*n* = 12): Control group, AOM/DSS group and EVO group. AOM/DSS and EVO groups mice were received a i.p. injection of 10 mg/ kg AOM and 7 days later, 2.5%DSS administration was repeated three times for whole test periods (Weeks 2, 5, and 8). From the first day of the model induction, the mice of EVO group were gavaged with 40 mg/ kg Evodiamine, Control group and AOM/DSS group received orally 0.5%CMC-Na respectively daily for 12 weeks (Figure 3A). During the study, body weights were measured every 3 days. Feces samples were collected on 84^th^ days and stored at −80°C for further analysis.

The mice were euthanized, their colons were measured and quantified the gross tumors (Tumor size (mm) = diameter of tumor). 1.0–2.0 cm sections were dissected from distal-colon and either snap-forzen in liquid N_2_ for futher analysis, stored in RNA later for RNA extraction, or fixed in 4%paraformaldehyde for histology assessment. All animal experiments were approved by the institutional animal care and use committee and conducted in accordance with Care and Use of Laboratory Animals guidelines.

### Histopathological Analysis

After fixation for 48 h by 4%paraformaldehyde, the formalin-fixed colon samples were buried in paraffin wax and 3-μm thick paraffin sections were stained with hematoxylin-eosin (HE) for histological analysis. Tissue pathology was scoring using previously described systems (Ranganathan et al., 2013). And images were measured under the microscope (Leica DM5000B, Leica, Germany).

### Alcian Blue-Periodic Acid Sthiff Staining

To analyze goblet cells (GCs), the fixed colons were then stained using Alcian blue and periodic acid-Schiff (AB-PAS). Observation and taking photos were performed under standing microscope (BX53, Olympus, Japan). GCs were quantified using the Cell counter Image-Pro Plus 6.0 software and normalized to crypt numbers, the analysis was performed on 10 crypts per animal.Frontiers requires figures to be submitted individually, in the same order as they are referred to in the manuscript.

### Terminal Deoxynucleotidyl Transferase dUTP Nick End Labeling Staining

Apoptosis was detected by a terminal deoxynucleotidyl transferase dUTP nick end labeling (TUNEL) according to the manufacturer’s protocol (Wanleibio). Images were visualized by Confocal Scanning Laser Microscope (ZEISS, Germany). We randomly selected five tumor sections for TUNEL detection, and TUNEL-positive cells were counted in images by Image-Pro Plus 6.0.

### Immunohistochemistry Staining

Paraffin-embedded tissues were used for analyzing the expression of Ki-67. Tissue sections were deparaffinised and rehydrated using a graded ethanol series and distilled water, and then treated with 3%H_2_O_2_ in methanol for 30 min then washed with PBS, 10%normal goat serum incubated for 30 min. After washing, primary antibody Ki-67 (ab16667, 1:300) was applied to tissue overnight at 4°C. Sections were then washed in PBS three times and incubated with secondary antibodies. Following washing, sections were developed with DAB using a commercial kit (CoWin Biosciences), counterstained with hematoxylin, coverslipped. Finally, observation and taking photos were performed under standing microscope (BX53, Olympus, Japan). The proportion of Ki-67 positive cells was determined by counting immunostain positive cells, as a percentage to the total number of nuclei in the field. At least 1,000 cells were counted in five random microscopic fields, which was analysed by Image-Pro Plus 6.0.

### Colon RNA Extraction and Real-Time PCR

Total RNA was extracted from colon tissues using Trizol reagent (TaKaRa) and quantified for cDNA synthesis. Quantitative real-time PCR reactions were performed using the CFX96 detection system (Bio-Plex) and SYBR Green (TIANGEN). The primers are listed in Supplementary Table S2. The expression levels were calculated with the 2^−ΔΔCt^ method, and the Ct values were normalized using GAPDH as a reference gene.

### 16S rRNA Amplicon Sequencing

Feces on day 18 and 84 were used to detect changes in the gut microbiota of the mice. Bacterial DNA in feces was extracted by the Stool Genomic DNA Kit (QIAGEN, Germany) then amplified by qPCR using the primers: 338F (5′-ACT​CCT​ACG​GGA​GGC​AGC​AG-3′) and 806R (5′-GGACTACHVGGGTWTCTAAT-3′). All samples were sequenced using the 250-base pair paired-end strategy on the Illumina HiSeq 2,500 platform according to the manufacturer’s instruction. High-quality sequence reads were then analyzed using the Quantitative Insights Into Microbial Ecology (QIIME, 1.9.1, http://qiime.org/scripts/assign_taxonomy.html) software package and visualized using R software (Version 3.4.1). Functional contributions of the microbial communities were predicted based on OUT using R package Tax4Fun with the Kyoto Encyclopedia of Genes and Genomes (KEGG) database (Asshauer et al., 2015).

### Liquid Chromatography-Mass Spectrometry-Based Non-targeted Metabolomics Analyses

50 mg of feces was collected into a tube and added methanol-water (4:1, v/v), crushed by a high-throughput tissue crusher at low temperatures; vortexing and three cycles of ultrasound (10 min); placed at −20°C for 30 min; and centrifuged at 13,000 × g and 4°C for 15 min. The supernatants were subjected to metabolomics profiling. A quality control (QC) sample, made by mixing and blending equal volumes (10 μL) of each sample, was used to examine the entire analysis process instrument stability and ensure the reliability of the results.

LC-MS was performed on a Thermo UHPLC system equipped with a binary solvent delivery manager and a sample manger, coupled with a Thermo Q Exactive Mass Spectrometer equipped with an electrospray interface. LC conditions: Column: Acquity BEH C18 column (100 mm × 2.1 mm i.d., 1.7 μm; Waters, Milford, United States). Solvent: Solvent A was aqueous formic acid (0.1% (v/v) formic acid) and Solvent B was acetonitrile/isopropanol (1/1) (0.1% (v/v) formic acid). The gradient was set as follows: 0–3 min, 5%B; 3–9 min, 20%B; 9–13 min, 95%B; 13.0–13.1 min, 95%B; 13.1–16 min, 5%B. Injection volume was 10 μL, flow rate was 0.4 ml/ min, and column temperature was set at 40°C. The mass spectrometric data was collected using a Thermo UHPLC-Q Exactive Mass Spectrometer equipped with electrospray ionization (ESI) source operating in either positive or negative ion mode.

The total ion chromatograms were collected and the ion selection, peak alignment, peak matching and filtering was carried out by Progenesis QI (Waters Corporation, Milford, United States). The online METLIN database (https://metlin.scripps.edu/) and HMDB database (http://www.hmdb.ca/) were used to identify metabolites. After normalization, data were exported to SIMCA V13.0 (Umetrics, Sweden) for multivariate data analysis. The differential compounds were screened by q value (p value adjusted by false discovery rate (FDR))≥0.05 and variable importance in the projection (VIP)≥1 from the cross-validated PLS-DA model. Finally, the biomarkers were analyzed by Metabolomics Pathway Analysis (MetPA) to describe the involved potential metabolic pathways.

### Colon Transcriptomics

Total RNA was extracted from colon tissue using TRIzol^®^ Reagent according the manufacturer’s instructions (Invitrogen) and genomic DNA was removed using DNase I (TaKara). Extract RNA, gene expression and data analysis was performed as our previous study (Wang MX. et al., 2020).

### Statistical Analysis

The correlation between microbes and physiological index was calculated by Spearman correlation with SPSS (version 21.0). All the values in the tables and figures are expressed as the mean ± SD. Statistical analyses were carried out using t-test between two groups and one-way ANOVA followed by Tukey-Kramer post-hoc test among multiple groups using SPSS software. A difference of *p* < 0.05 was considered as statistical significance.

## Results

### EVO Suppressed AOM/DSS-Induced Colitis in Mice

To determine whether EVO could attenuate the acute colitis of CAC, AOM-induced mice were challenged with 2.5%DSS for 7 days, and EVO was gavaged throughout the experiment (Figure 1A). Disease activity index (DAI) score, including body weight loss, presence of blood in stool and diarrhea, were observed after the DSS challenge (Figure 1C). Treatment with EVO relieved the body weight loss and decreased the DAI score (Figures 1B,C). In addition, the shortened colon length induced by DSS was significantly increased by EVO administration (Figures 1D,E). HE staining showed that AOM/DSS induced the destruction of intestinal epithelial structure, massive infiltration of inflammatory cells, crypt loss and large area edema (Figure 1G), accompanied by an increase in pathological score (Figure 1F). However, these pathological changes were remarkably reversed by EVO treatment (Figures 1F,G). EVO treatment also significantly inhibited the concentration of pro-inflammatory cytokines TNF-α and IL-1β, increased the expression of anti-inflammatory cytokine IL-10 (Figure 1H). Collectively, these results suggested that EVO ameliorated the acute colitis of CAC mice.

### EVO Restored Intestinal Barrier Function in AOM/DSS-Induce Colitis in Mice

**FIGURE 2:**
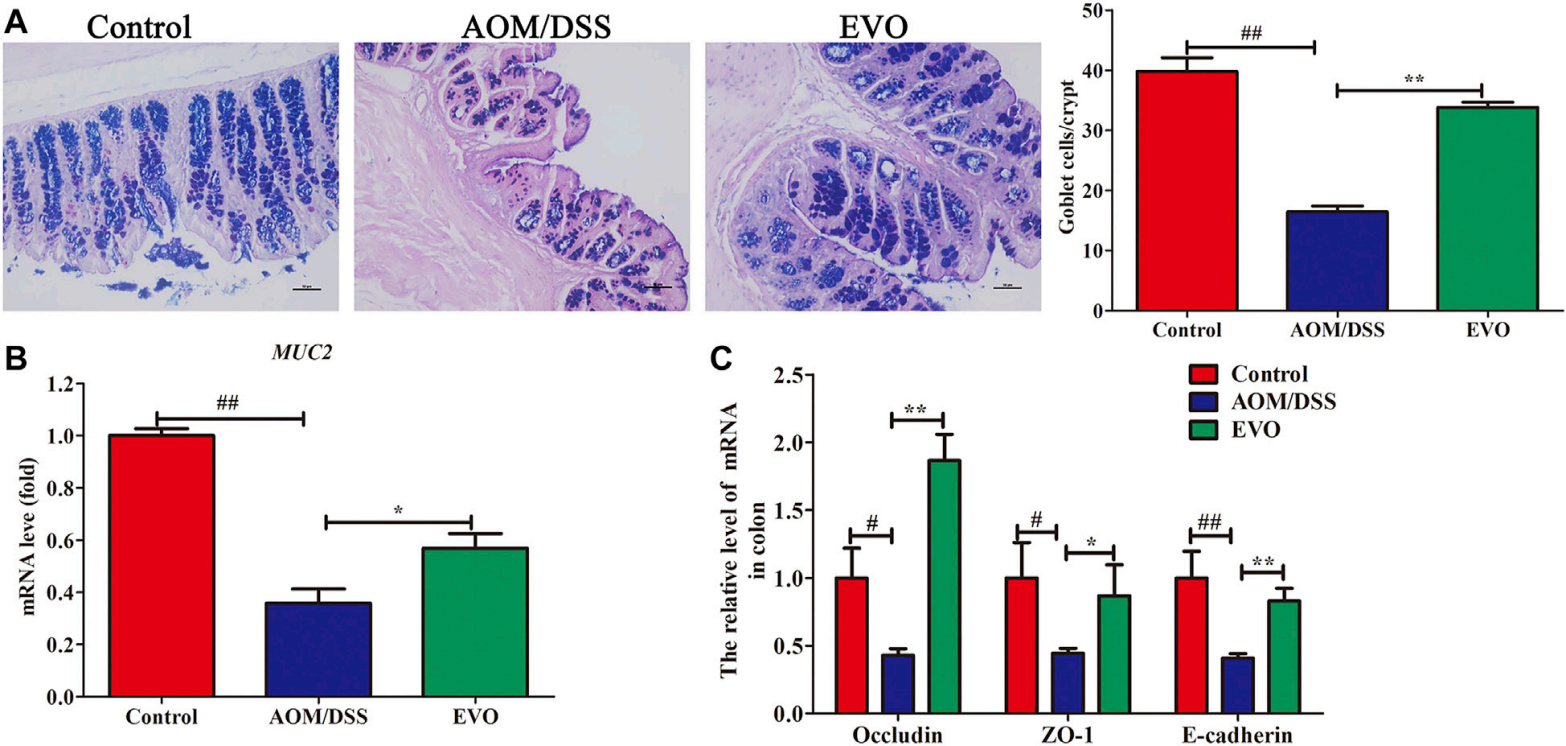
EVO improves damage to the colonic mucosal barrier in AOM/DSS-induced colitis mice. **(A)** AB-PAS staining and the number of goblet cells. **(B)** The relative expression of *MUC2*. **(C)** Changes in colon mucosal barrier molecule mRNA levels. The results are presented as mean ± SD. ^#^ *p <* 0.05, ^##^ *p <* 0.01, Control *vs*. AOM/DSS; ^*^ *p <* 0.05, ^**^ *p <* 0.01, EVO *vs*. AOM/DSS.

We measured AB-PAS staining, MUC2 and tight junction molecules (Occludin, ZO-1 and E-cadherin) in the colon as parameters for the integrity of intestinal barrier. AB-PAS staining of the gut tissues of representative mice in each group showed that the quantity of goblet cells in AOM/DSS group was significantly reduced (Figure 2A). EVO supplementation significantly increased the number of goblet cells, promoted the secretion of MUC2 and the expression of Occludin, ZO-1 and E-cadherin (Figures 2A–C). These results indicated that EVO could rescue these AOM/DSS-induced damage to the gut barrier and increase gut permeability.

### EVO Attenuated AOM/DSS-Induced Colon Tumorigenesis in CAC Mice

**FIGURE 3:**
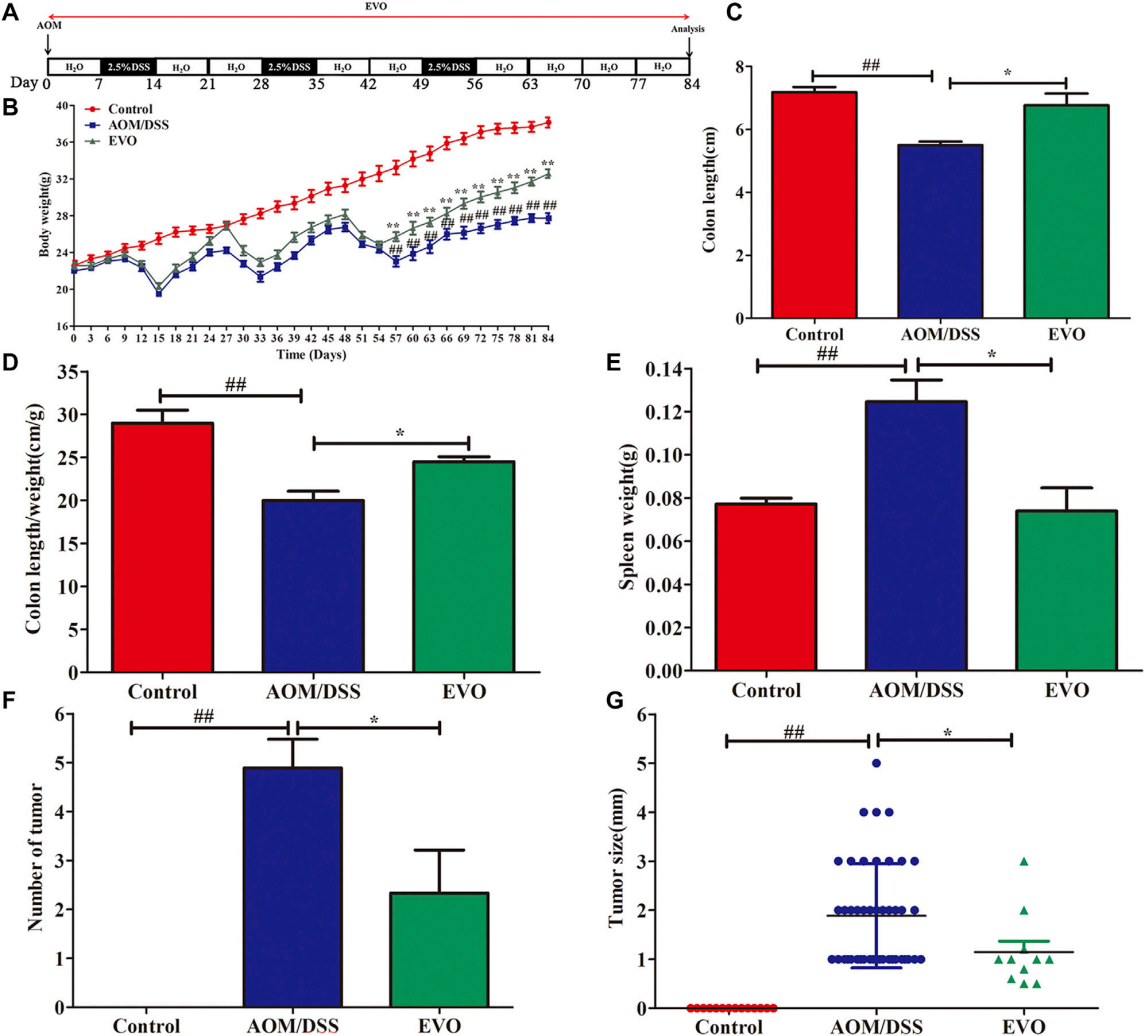
EVO prevents tumorigenesis and growth at a late stage of CAC. **(A)** Protocol of EVO and AOM/DSS treatments used in this study. **(B)** Body weight change. **(C)** Colon length. **(D)** Colon length/weight ratio. **(E)** Spleen weight. **(F)** Tumor number. **(G)** Tumor size. The results are presented as mean ± SD. ^#^ *p <* 0.05, ^##^ *p <* 0.01, Control *vs*. AOM/DSS; ^*^ *p <* 0.05, ^**^ *p <* 0.01, EVO *vs*. AOM/DSS.

**FIGURE 4:**
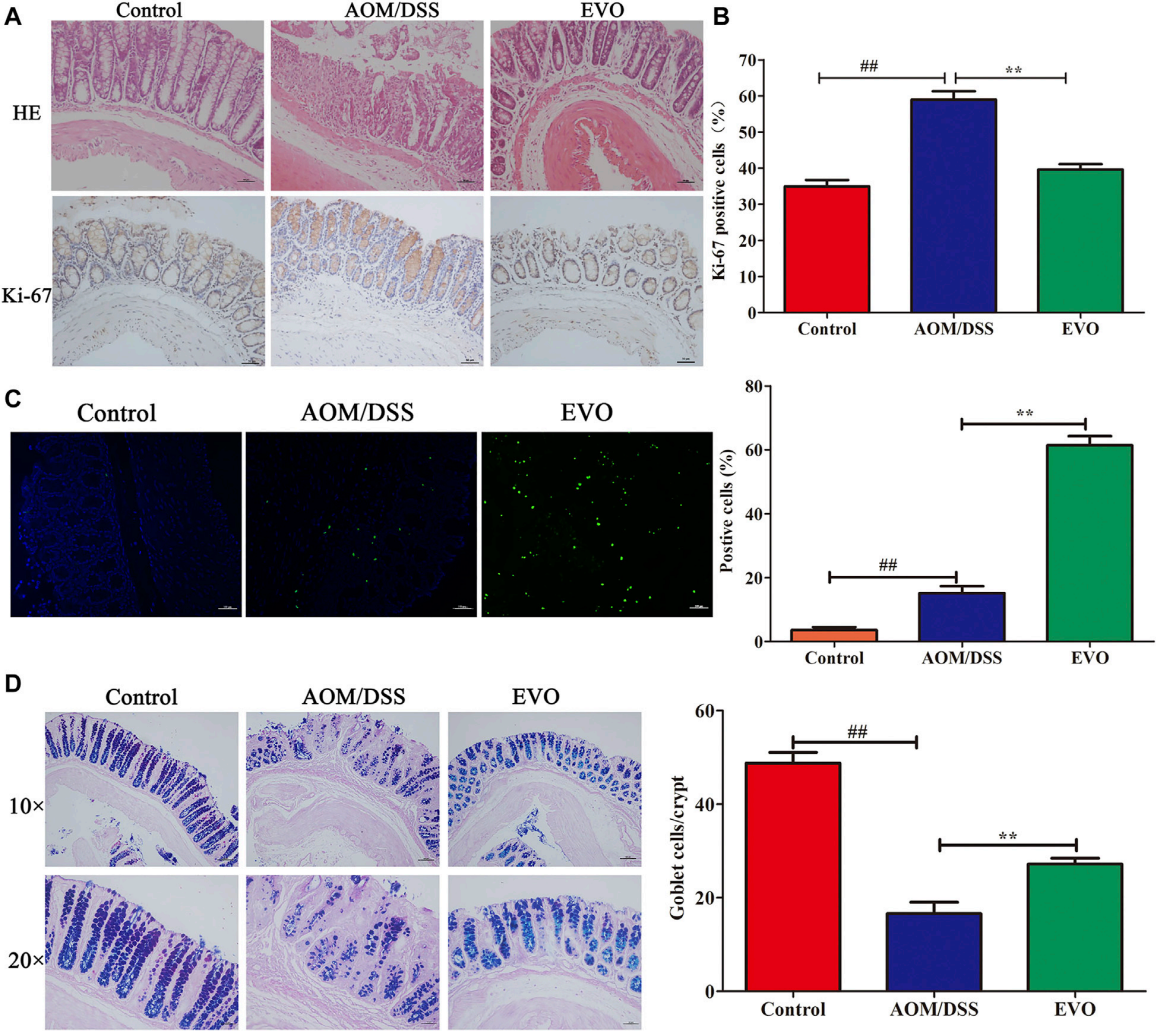
| Effect of EVO treatment on cell proliferation and apoptosis in CAC model. **(A)** HE staining and Ki-67 analysis. **(B)** The proportion of Ki-67 positive cells in the colon of CAC mice. **(C)** Representative photo for TUNEL staining and assessment of the apoptotic cells. **(D)** AB-PAS staining and the number of goblet cells. The results are presented as mean ± SD. ^##^ *p <* 0.01, Control *vs*. AOM/DSS; ^**^ *p <* 0.01, EVO *vs*. AOM/DSS.

To investigate the impact of EVO on the intestinal tumor, we established AOM/DSS recurring inflammation-driven colorectal cancer (Figure 3A). AOM/DSS administration reduced the body weight, however, EVO group showed a protective effect on weight loss (Figure 3B). Expectedly, EVO treatment could significantly increase the colon length and the length-weight ratio of colon, and relieved splenomegaly (Figures 3C–E). All mice in the AOM/DSS group had colon tumors, but administration with EVO suppressed tumor cells development and decreased the tumors size (Figures 3F,G). In the AOM/DSS group, high-grade dysplasia of intestinal epithelium and heavily infiltrated with inflammatory cells were observed, indicating the existence of inflammation and highly differentiated adenocarcinoma of the colon (Figure 4A). Treatment with EVO could inhibit inflammatory cells infiltration and tumorigenesis, and maintain the intestinal epithelial structure. To investigate the effect of EVO on tumor cells, we examined the proliferation and apoptosis in colon tumor tissues. The rate of Ki-67 positive cells in AOM/DSS was significantly increased than that in EVO group (Figures 4A,B). Compared with the AOM/DSS group, the number of apoptotic cells increased in the EVO group (Figure 4C). These results suggested that EVO inhibited the proliferation of colon tumor cells and promoted their apoptosis. Finally, we used AB/PAS staining to detect the goblet cells quantity. The results showed that the numbers of mucin granule-containing goblet cells were markedly lower after exposure to AOM/DSS than in the EVO group (Figure 4D). The result indicated that EVO protected the mucous layer and thus inhibited the tumorigenesis.

### EVO Treatment Modulated the Composition of Gut Microbiota in AOM/DSS-Induced Colitis Mice

**FIGURE 5:**
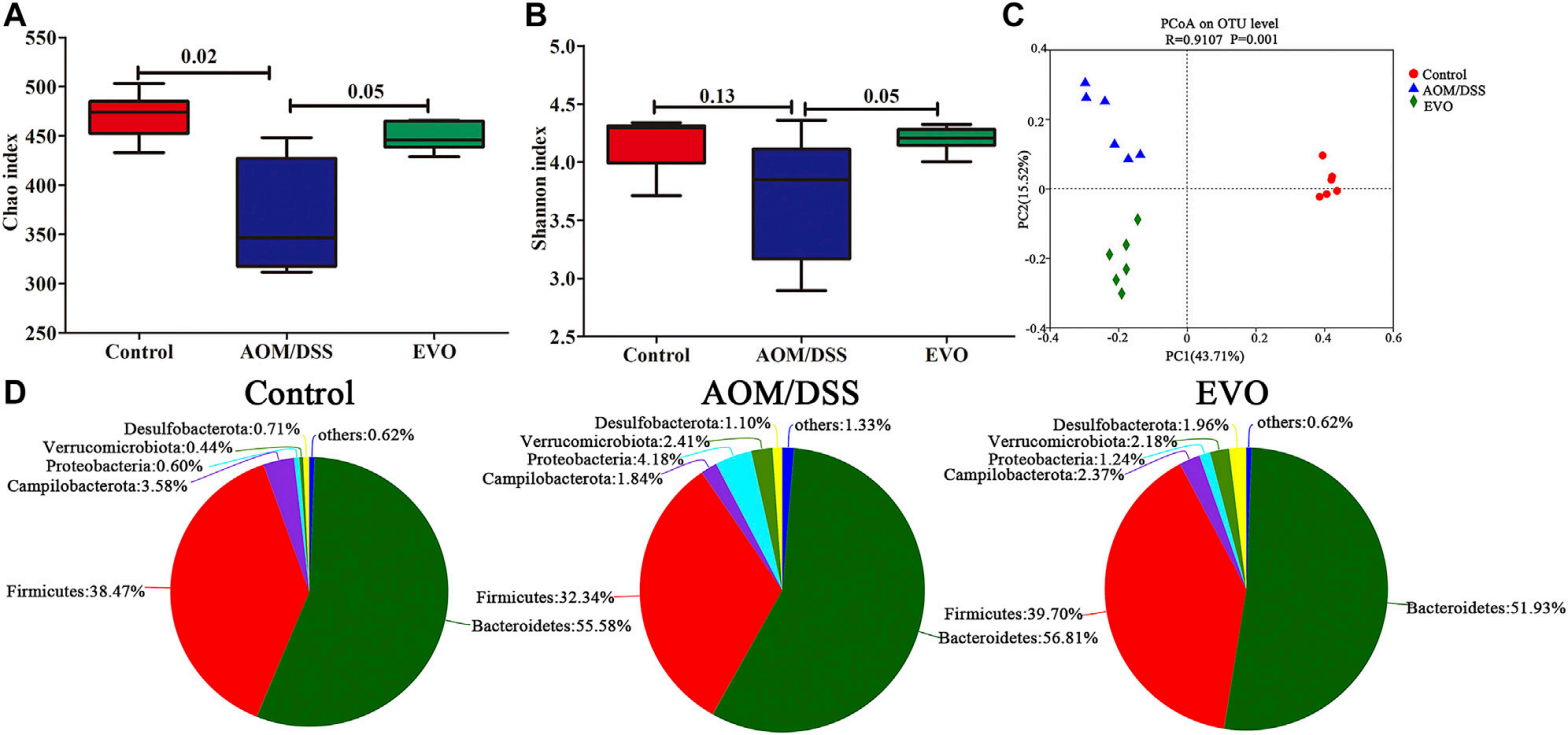
| EVO alters AOM/DSS-induced colitis mice fecal microbiota composition. **(A)** Chao index. **(B)** Shannon index. **(C)** UniFrac-based PCoA plot. **(D)** Phylum levels pie chart.

To investigate the impact of EVO on gut microbiota composition in AOM/DSS-induced colitis mice, we performed 16S rRNA gene sequencing of feces in three groups. The α-diversity indices, richness indices (Ace and Chao) and diversity indices (PD whole tree, Shannon and Simpson) were declined in AOM/DSS group compared with Control group, and EVO treatment could impact on the α-diversity of microbiota (Figures 5A,B; Supplementary Figure S1A–C; Supplementary Table S3). To characterize the global differences in feces microbial communities among groups, a principal coordinates analysis (PCoA) was performed on bray_curtis. The PCoA plots showed a distinct clustering of the microbiota composition among the three groups (analysis of similarity [ANOSIM]; between Control group and AOM/DSS group, *p* = 0.003; between AOM/DSS group and EVO group, *p* = 0.007), indicating EVO could modulate the structure of gut microbiota in AOM/DSS-induced colitis mice (Figure 5C; Supplementary Figure S1D–E).

**FIGURE 6:**
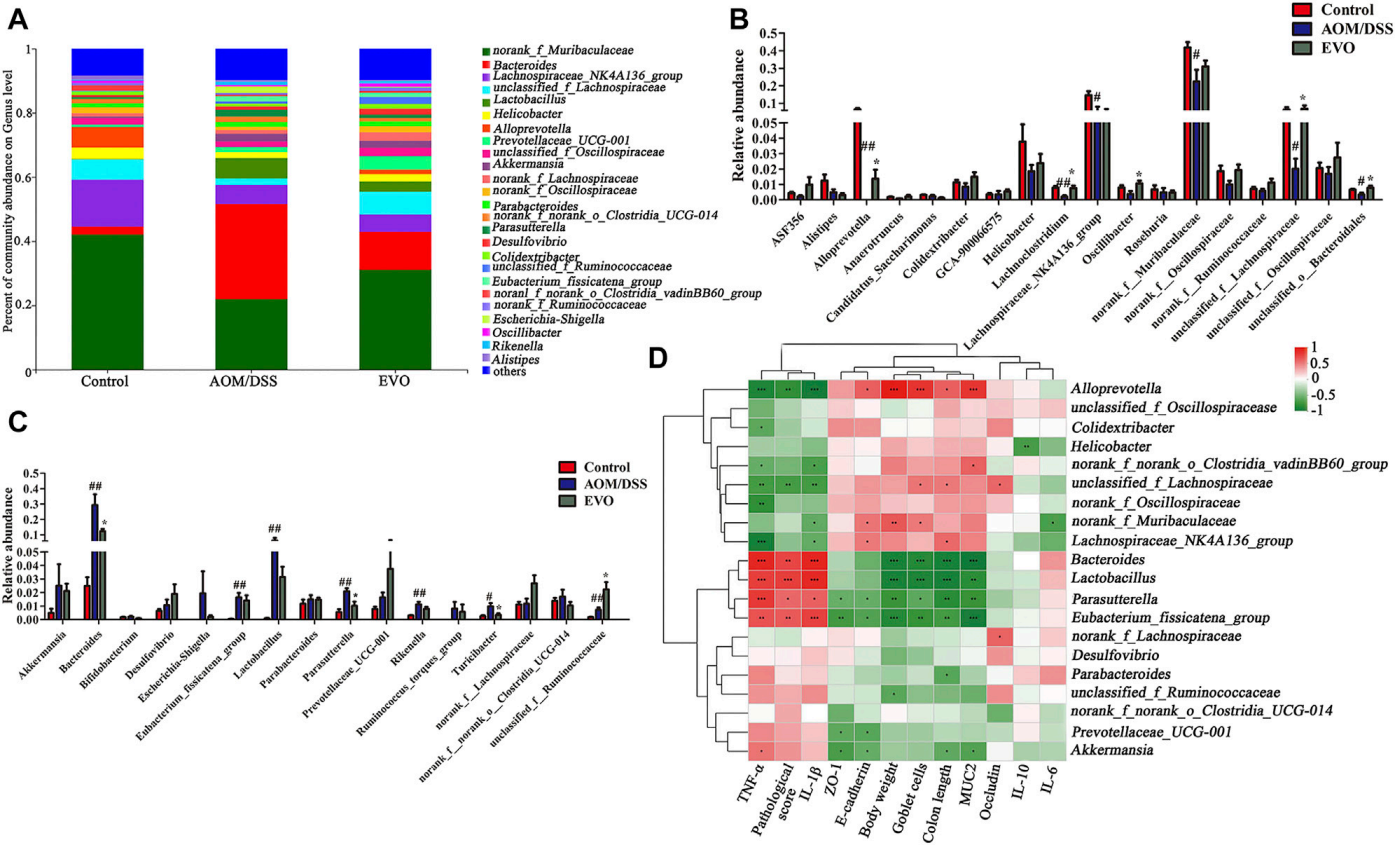
| Analysis of gut microbiota following EVO treatment in AOM/DSS-induced colitis mice. **(A)** The intestinal microbiota distribution at the family level. The up-regulated gut microbiota **(B)** and the down-regulated gut microbiota **(C)** at the genus level. **(D)** Spearman’s correlation analysis of microbiota and physiological indexes. ^*^ *p <* 0.05, ^**^ *p <* 0.01, ^***^ *p <* 0.001.

To investigate the abundance and distribution of gut microbiota in the Control group, AOM/DSS group and EVO group, we analyzed the variation of microbiota at the phylum level. Six primary phyla (Bacteroidetes, Firmicutes, Campilobacterota, Desulfobacterota, Proteobacteria and Verrucomicrobiota) were shown in the bacterial communities (Supplementary Table S4). Compared with Control group, AOM/DSS-induced the relative abundance of Firmicutes and Campilobacterota were decreased, and the levels of Bacteroidetes, Desulfobacterota, Proteobacteria and Verrucomicrobiota were elevated, while it was restored by EVO treatment (Figure 5D; Supplementary Figure S1F). Moreover, the Firmicutes/Bacteroidetes ratio increased from 0.72 to 0.81 by EVO supplementation (Supplementary Figure S1G). To further explore the changes of gut microbiota community in each group, an analysis of the genus level was performed (Figure 6A). Based on a 97% species similarity, a total of 135 genera were identified from the three groups, of which 34 genera with relative abundance made up at least more than 0.5% in one group were analyzed. Compared with the Control group, the relative abundance of *ASF356*, *Alistipes*, *Alloprevetella*, *Anaerotruncus*, *Candidatus_Saccharimonas*, *Colidextrribacter*, *GCA-900066575*, *Helicobacter*, *Lachoclostridium*, *Lachnospiraceae_NK4A136_group*, *Oscillibacter*, *Roseburia*, *norank_f_*Muribaculaceae, *norank_f_Oscillospiraceae*, *norank_f_Ruminococcaceae*, *unclassified_f_Lachnospiracease*, *unclassified_f_Oscillospiraceae*, and *unclassified_o_Bacteroidales* were decreased, and the levels of *Akkermansia*, *Bacteroides*, *Bifidobacterium*, *Desulfovibrio*, *Escherichia-Shigella*, *Eubacterium_fissicatena_group*, *Lactobacillus*, *Parabacteroides*, *Parasutterella*, *Prevotellaceae_UCG-001*, *Rikenella*, *Ruminococcus_torques_group*, *Turicibacter*, *norank_f__norank_o__Clostridia_UCG-014*, *unclassified_f__Ruminococcaceae* were increased (Figures 6B,C). However, EVO treatment could significantly increase the relative abundance of *Alloprevotella*, *Lachnoclostridium*, *Oscillibacter*, u*nclassified_f_Lachnospiraceae*, *unclassified_f_Ruminococcaceae*, *unclassified_o_Bacteroidales*, and decrease the levels of *Bacteroides*, *Parasutterella*, *Turicibacter*. Taken together, our results showed that EVO treatment changed the composition of gut microbiota after AOM/DSS-induced colitis mice.

### Association Between Gut Microbiota and Colitis-Related Pathophysiological Traits

To further investigate whether changes in gut microbiota are correlated with the remission of colitis symptoms, the Spearman correlation between the top 20 genus and related physiological indicators among three groups (Figure 6D, Supplementary Table S5). We found that the feces microbiota (*Bacteroides*, *Lactobacillus*, *Parasutterella* and *Eubacterium_fissicatena_group*; *p <* 0.05) were negative correlation with body weight, goblet cells, colon length and MUC2, and were positive correlation with TNF-α, pathological score and IL-1β, while the correlation between *Alloprevotella* and indicators were just opposite to the above results. Interestingly, we also found that *Akkermansia* was negative associated with MUC2、colon length、ZO-1 and E-cadherin, was positive associated with TNF-α (*p <* 0.05), *Lachnospiraceae_NK4A136_group* was positive correlation with colon length and E-cadherin, was negative correlation with TNF-α and IL-1β (*p <* 0.05).

### The Effect of EVO on Gut Microbiota in CAC Mice

**FIGURE 7:**
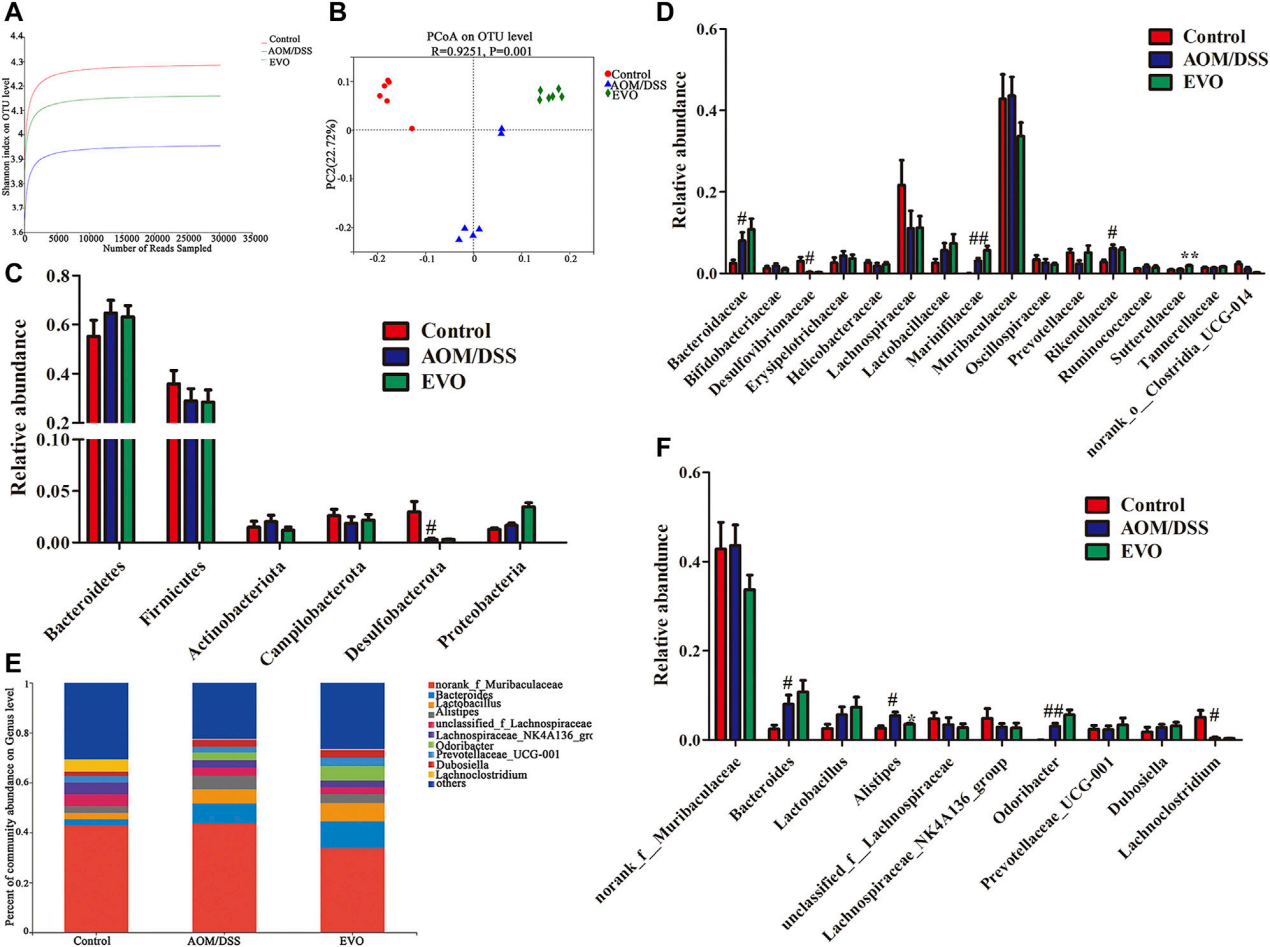
| EVO changes the gut microbiota of AOM/DSS-induced colon cancer mice. **(A)** Shannon index. **(B)** UniFrac-based PCoA plot. The relative abundance of gut microbiota at Phylum level **(C)** and at the family level **(D)**. **(E–F)** The relative abundance of gut microbiota at genus level. ^#^ *p <* 0.05, ^##^ *p <* 0.01, Control *vs*. AOM/DSS; ^*^ *p <* 0.05, ^**^ *p <* 0.01, EVO *vs*. AOM/DSS.

To further investigate whether EVO attenuated AOM/DSS-induced colon tumorigenesis is related to gut microbiota, 16S rRNA analysis was performed on feces samples collected at 84^th^ days. Common microbial α-diversity indices, richness indices (Chao and Ace) and diversity indices (Shannon, Simpson and PD whole tree) were declined in AOM/DSS group, and EVO treatment could increase the diversity indices and not change the richness indices, indicating that EVO can increase microbial community diversity (Figure 7A; Supplementary Figure S2A–E; Supplementary Table S6). PCoA based on the Unweighted UniFrac algorithm clearly revealed that the microbial community changed among three groups (Figure 7B). To further understand the result of PCoA, the Hierarchical clustering analysis was conducted, the Hierarchical clustering tree showed an obvious separation among three groups (Supplementary Figure S2F).

Based on the average abundance analysis, at the phylum level, Bacteroidetes, Firmicutes, Actinobacteria, Campilobacterota, Desulfobacterota and Proteobacteria were the major phyla in the three groups. Compared with Control group, the relative abundance of Bacteroidetes, Actinobacteria and Proteobacteria were increased, and Firmicutes, Campilobacterota and Desulfobacterota were decreased in AOM/DSS group (Figure 7C). However, there was no significant difference in the relative abundance at the phylum level between EVO group and AOM/DSS group. At the family level, a total of 16 families with a relative abundance of more than 1% in at least one group were identified from the three groups (Figure 7D). AOM/DSS increased the relative abundance of *Bacteroidaceae*, Marinifilaceae and Rikenellaceae, and decreased the level of Desulfovibrionaceae (*p <* 0.05). Further, we observed that EVO markedly increased the relative abundance of Sutterellaceae (*p <* 0.05) and Lactobacillaceae (*p >* 0.05). To analyze the changes of gut microbiota at the genus level, we selected the ten must abundant microbial genera and their abundance was analyzed (Figure 7E). The most abundant genera were *norank_f_*Muribaculaceae, *Bacteroides*, *Lactobacillus*, *Lachnosirpacear_NK4A136_group* and *Prevotellacear_UCG-001*. The abundance of *Bacteroides*, *Alistipes* and *Odoribacter* were higher in the AOM/DSS group than in the Control group (Figure 7F). Compared with AOM/DSS group, the abundance of *Bacteroides*, *Lactobacillus*, Lachospiraceae*_NK4A136_group* and *Prevotellaceae_UCG-001* were higher in the EVO group (*p >* 0.05). It’s worth noting that EVO could significantly decrease the abundance of *Alistipes* (Figure 7F).

### Key Bacteria Responding to EVO Supplementation From Colitis to Tumors in CAC Mice

**FIGURE 8:**
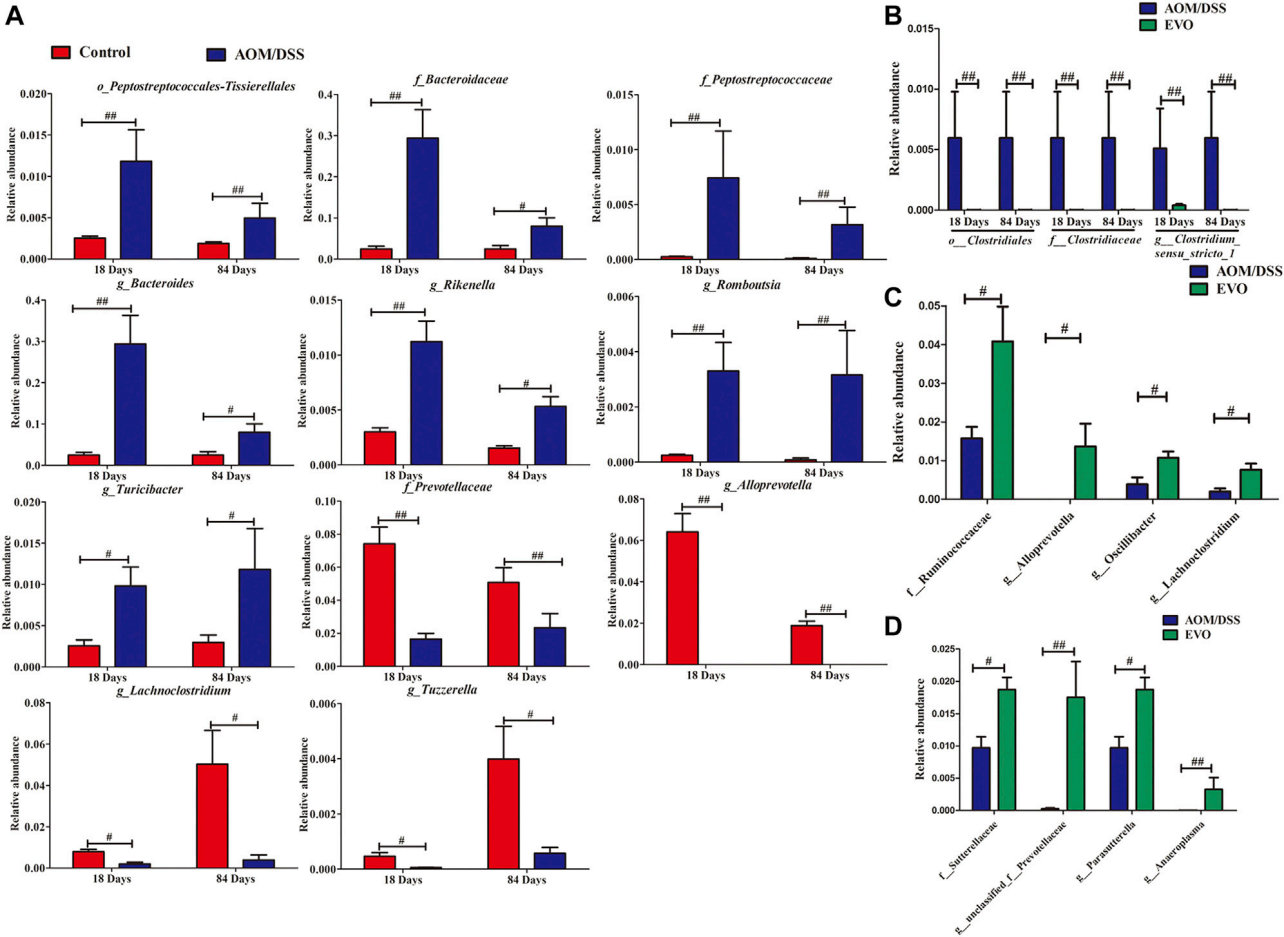
Taxonomic differences of gut microbiota among Control, AOM/DSS and EVO groups from colitis to tumorigenesis. **(A)** Comparison of the bacterial community in 18- and 84-days CAC mice in the Control group and AOM/DSS group. **(B)** Comparison of the bacterial community in 18- and 84-days CAC mice in the EVO group and AOM/DSS group. Comparison of the bacterial community in 18-days **(C)** and 84-days **(D)** CAC mice in the EVO group and AOM/DSS group. ^#^ *p <* 0.05, ^##^ *p <* 0.01.

We jointly analyzed the microbiota evolution of CAC mice at 18 and 84 days by using LEfSe to identify the specific bacterial phylotypes that were changed by EVO. On the basis of taxa with linear discriminant analysis (LDA) scores greater than three and *p <* 0.05, we concluded that CAC model mice, compared with Control group altered 68 (50 increased and 18 decreased), and 42 (19 increased and 23 decreased), at 18 and 84 days respectively (Supplementary Figures S3A, S3C). We can see that the harmful bacteria (e.g., *g_Rikenella, g_*Streptococcaceae、*g_*Erysipelatoclostridiaceae and *g_Mucisirillum*) increased significantly in the early stage of colitis, while the beneficial bacteria (e.g., *g_Lachnoclostridium, g_Prevotellaceae_NK3B31_group, g_Candidatus_Arthromitus* and *g_Alloprevotella*) decreased significantly in the later stage of tumor. Among the 69 increased bacterial genera, CAC model mice altered 7 shared genera from colitis to tumor, and while in 41 reduced genera, CAC model mice altered 4 shared genera (Figure 8A). Levels of *o__Peptostreptococcales-Tissierellales, f__Bacteroidaceae, f__*Peptostreptococcaceae*, g__Bacteroides*、*g__Rikenella, g__Romboutsia* and *g__Turicibacter* were significantly enriched in CAC mice, whereas *f__Prevotellaceae, g__Alloprevotella, g__Lachnoclostridium* and *g__Tuzzerella* were reduced observably in the AOM/DSS group compared with Control group.

And treatment with EVO, compared with CAC model mice altered 28 (9 increased and 19 decreased), and 27 (20 increased and 7 decreased), at 18 and 84 days respectively (Supplementary Figures S3B, S3D). We also can see that treatment with EVO could decrease significantly the harmful bacteria (e.g., *f_*Erysipelotrichaceae*, f_*Deferribacteraceae*, g_Mucispirillum* and *g_Streptococcus*) in the early stage of colitis, while increase the beneficial bacteria (e.g. *f__*Sutterellaceae、*g__unclassified_f__Prevotellaceae, g__Parasutterella* and *g__Anaeroplasma*) in the later stage of tumor. Among the 26 reduced bacterial genera, EVO treatment altered three shared genera from colitis to tumor (Figure 8B). Levels of *o__Clostridiales, f__Clostridiaceae* and *g__Clostridium_sensu_stricto_1* were significantly reduced in EVO group compared with AOM/DSS group. And while in 29 increased genera, EVO treatment altered 0 shared genera, indicating that different probiotics may play a role in different stages of CAC. In the early stage of colitis, EVO could increase the levels of *f__Ruminococcaceae*、*g__Alloprevotella, g__Oscillibacter* and *g__Lachnoclostridium* (Figure 8C). Levels of *f__*Sutterellaceae、*g__unclassified_f__Prevotellaceae, g__Parasutterella* and *g__Anaeroplasma* were enriched significantly in EVO group at the late stage of CAC(Figure 8D).

### Predictive Microbiota Functional Profiling in CAC Mice

**FIGURE 9:**
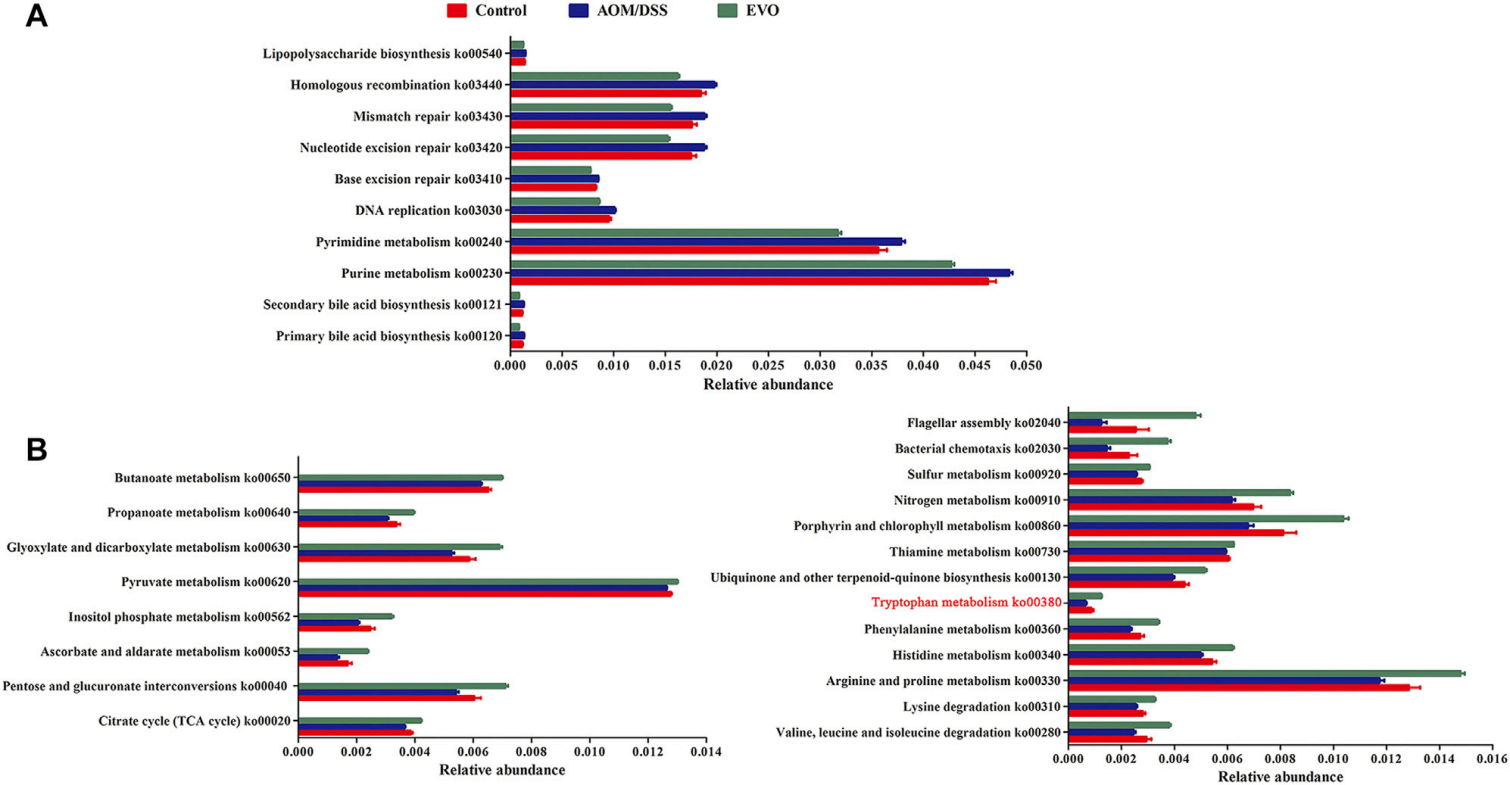
Interred gut microbiota functions by Tax4Fun from 16S rRNA gene sequences among Control, AOM/DSS and EVO groups in AOM/DSS-induced colon cancer mice. **(A)** Up-regulated metabolism and down-regulated metabolism **(B)** in EVO group.

Tax4Fun based on OTUs was used to predict the functional contribution of the microbiota. We identified 4975 Kyoto Encyclopedia of Genes and Genomes (KEGG) orthologs (KOs), mainly belonging to the pathway Metabolism, Environmental Information Processing and Genetic Information Processing. A total of 2,796 KOs were identified with significantly different abundances between AOM/DSS group and Control group, and 3592 KOs differed in abundance between EVO group and AOM/DSS group (false-discovery rate [FDR], *p <* 0.05). In the level 3 KEGG pathways, the microbial gene functions, including those associated with lipid metabolism (ko00120、ko00121), nucleotide metabolism (ko00230, ko00240), replication and repair (ko03030, ko03410, ko03420, ko03430, ko03440) and Glycan biosynthesis and metabolism (ko00540), were decreased significantly in EVO group, while those carbohydrate metabolism (ko00020, ko00040, ko00053, ko00562, ko00620, ko00630, ko00640, ko00650), amino acid metabolism (ko00280, ko00310, ko00330, ko00340, ko00360, ko00380), metabolism of cofactors and vitamins (ko00130, ko00730, ko00860), energy metabolism (ko00910、ko00920) and cell motility (ko02030, ko02040) were increased (FDR, *p <* 0.05, Figures 9A,B).

### EVO Regulated Metabolites in AOM/DSS-Induced Colon Cancer Mice

**FIGURE 10:**
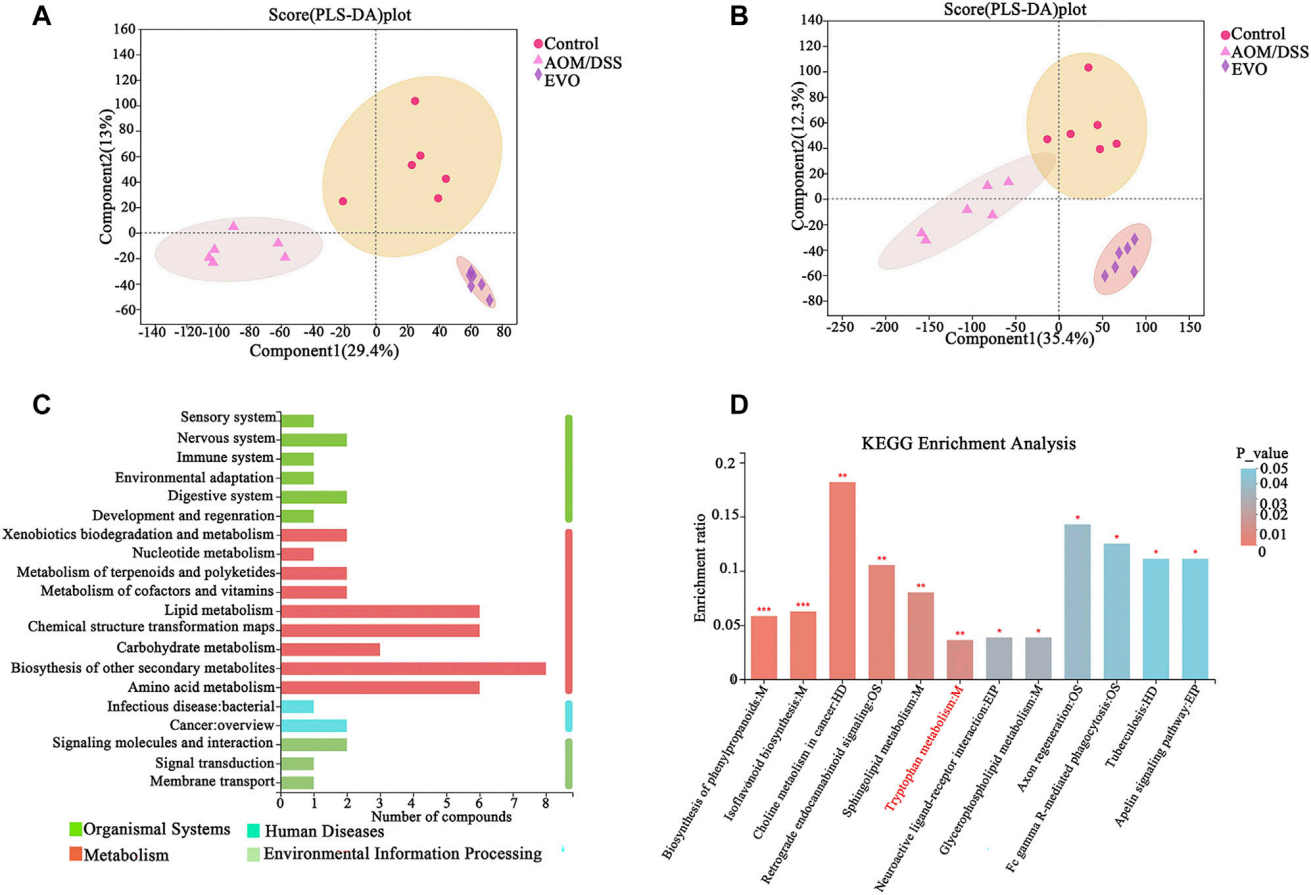
The difference of fecal metabolites among Control, AOM/DSS and EVO groups in AOM/DSS-induced colon cancer mice. PLS-DA map in positive ion model **(A)** and in negative ion model **(B)**. **(C)** KEGG pathway on level 1 and level 2 related to differential metabolites. **(D)** KEGG pathway enrichment column chart.

To investigate the effects of EVO on metabolite profiles CAC mice, we examined metabolites in the fecal in a non-targeted manner. The heat map of sample correlation showed a high similarity of metabolites expression in the samples within the group (Supplementary Figure S4A–B). PCA and PLS-DA were performed to identify the changes in metabolite profiles on positive and negative ESI data. PCA of metabolites showed a clear separation between AOM/DSS and Control groups, and overlaps existed between EVO and Control groups (Supplementary Figure S4C–D). The resulting score plot from PLS-DA in the positive (for the first four components, R^2^Y = 0.977 and Q^2^ = 0.836) and negative mode (for the first four components, R^2^Y = 0.978 and Q^2^ = 0.881) indicated a clear separation among the three groups (Figures 10A,B). In order to test the validity of the PLS-DA model, a permutation test (200 times) for PLS-DA was applied (Supplementary Figure S4E–F). The R^2^Y and Q^2^ values derived from the permuted data were lower than the original points to the right, indicating the validation of the PLS-DA model.

Multivariate statistics (VIP_OPLS-DA>1) and unit statistics (*p*_value *<* 0.05) were used to screen differential metabolites, a total of 355 differential metabolites were identified between AOM/DSS and Control groups, and 370 differential metabolites in abundance between EVO and AOM/DSS groups (Supplementary Tables S7, S8). Among these, 242 differential metabolites disturbed by AOM/DSS could be restored by EVO, we further analyzed 242 differential metabolites on the KEGG pathways and found that differential metabolites were mainly involved in biosynthesis of other secondary metabolites, lipid metabolism, chemical structure transformation maps and amino acid metabolism (Figure 10C). KEGG metabolic pathway enrichment analysis showed that the differential metabolites were mapped to about 41 pathways (Supplementary Table S9). The metabolites were significantly enriched in 12 KEGG pathways (*p*_value *<* 0.05), and biosynthesis of phenylpropanoids, isoflavonoid biosynthesis, choline metabolism in cancer, restrograde endocannabinoid signaling, sphingolipid metabolism and tryptophan metabolism were the six pathways with the most significant enrichment (Figure 10D). Based on the Tax4Fun function prediction and non-target metabolomics studies, tryptophan metabolism was found to be co-metabolism pathway, indicating that tryptophan metabolism may play an important role in the effect of EVO on intestinal inflammation and carcinogenesis.

### EVO Regulated the Gene Expressions of Colon Epithelial Cells in CAC Mice

**FIGURE 11:**
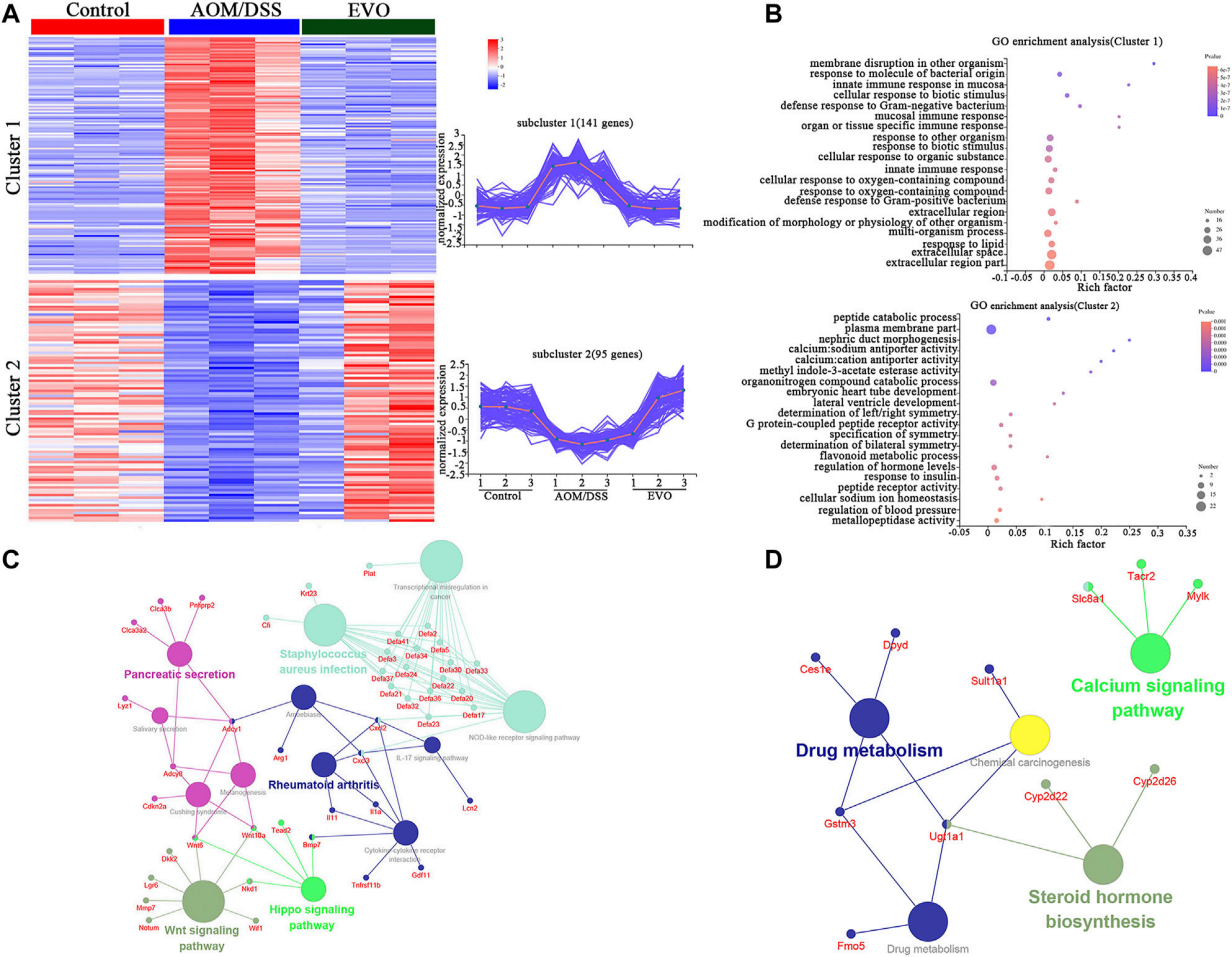
Comparisons of gene expression profiles by RNA-seq among Control, AOM/DSS and EVO groups in AOM/DSS-induced colon cancer mice in the colon. **(A)** Genewise clustering heatmap of all 236 differentially expressed genes according to the gene expression patterns, showing segregation into two clusters. **(B)** Doptplot of GO terms enriched for the two clusters. Down-regulating the genes **(C)** and up-regulating the genes **(D)** enrichment KEGG pathways.

The data above clearly showed that EVO could regulate the gut microbiota and metabolism of CAC mice. We performed RNA-Seq analysis to investigate the influence of EVO on gene expressions of colon epithelial cells in CAC mice. Compared with Control group, we obtained 863 up-regulated and 1421 down-regulated genes in the AOM/DSS group (*P*_adjust *<* 0.05, FC ≥ 2 or FC ≤ 0.5, Supplementary Table S10). However, 376 genes that were highly expressed and 280 genes that were lowly expressed in the EVO-treated group compared with AOM/DSS group (*P*_adjust *<* 0.05, FC ≥ 2 or FC ≤ 0.5, Supplementary Table S11). Furthermore, we identified 274 common genes in Control vs AOM/DSS and AOM/DSS vs EVO two data sets. Interestingly, 236 common genes showed an opposite trend of gene expression in AOM/DSS and EVO groups, indicating the involvement of these molecular targets in EVO-mediated prevention of CAC (Supplementary Table S12). Two clusters were obtained by clustering analysis of 236 differentially expressed genes according to their gene expression patterns in the three groups (Figure 11A). Then, GO enrichment analysis was performed on 236 genes belonging to two different clusters, and found that genes in Cluster 1 mainly enriched many GO terms related to immune response, such as “mucosal immune response” (*Defa2*, *Defa2*, 、*Defa30* etc.) and response to organism, such as “defense response to Gram-negative bacterium” (*Lyz1*, *Mmp7*, *Defa21* etc.) (Figure 11B). We found that differently genes in Cluster 1 were composed of significantly downregulated genes in the EVO group, suggesting a potential role of EVO therapy in reducing gut inflammation. In cluster 2, genes enriched some GO terms related to signal transduction, such as “methyl indole-3-acetate esterase activity” (*Ces1e*, *Ces1g*) and “G protein-coupled peptide receptor activity” (*Vipr2*, *Sstr1*, *Tacr2*, *Nlrp6*), and protein metabolism, such as “peptide catabolic process” (*Enpep*, *Cpe*, *Naaladl1*) and “peptide receptor activity” (*Vipr2*, *Sstr1*, *Tacr2*, *Nlrp6*) (Figure 11B). In view of the relatively high expression of genes in cluster two in EVO group, indicating that signal transduction and protein metabolism were significantly improved after EVO treatment. In the KEGG analysis, the downregulated genes were mainly concentrated in inflammatory signaling pathway (Wnt signaling pathway、Hippo signaling pathway、L-17 signaling pathway and Cytokine-cytokine receptor interaction), Pancreatic secretion and NOD-like receptor signaling pathway (Figure 11C). The upregulated genes were mainly enriched in Drug metabolism and Calcium signaling pathway (Figure 11D). Significant enrichment of functions and pathways may help us further investigate the role of differently genes in CAC. Together, our RNA-seq data reveal that EVO treatment significantly alleviates the inflammation of the CAC mice colon, potentially through the alteration in the gut microbiota.

## Discussion

Wu-Zhu-Yu, a longstanding Chinese botanical drug, is traditionally used for the treatment of digestive system disease, so it is important to understand how EVO inhibits intestinal inflammation and tumors. In this study, we evaluated the mechanism by which EVO relieved inflammation and inhibited the tumors in CAC mice through the gut microbiota, metabolic activity and transcription molecules. Collectively, according to the 16S rRNA sequencing, non-targeted metabolomics analyses and colon transcriptomics results, we revealed the mechanism of the effect of EVO on CAC mice from inflammation to colorectal tumors.

From the results above, we can see that EVO could significantly reduce the inflammation of AOM/DSS-induced colitis, which may be related to the maintenance of intestinal barrier function. The intestinal mucosal layer is the first line of defense against both commensal microbes and invading pathogens, the integrity of which is critical for health (Desai et al., 2016). The colonic mucus layer is a dynamic and chemically complex barrier, mainly composed of MUC2 secreted by GC, which forms the protective mucus barrier atop the intestinal epithelium (Allaire et al., 2018). Defects in the mucus layer increase susceptibility to pathogens, leading to low-grade inflammation and dysbiosis (Desai et al., 2016). The TJ plays an important role in maintaining the integrity of the intestinal barrier. The loss of barrier integrity increases the translocation of bacterial antigens and stimulates the inflammatory response of intestinal mucosa (Jin et al., 2021). In our results, the number of GC, the expression of MUC2 and tight junction protein in the gut tissues of CAC mice were increased by EVO. Inflammation is a critical link between IBD and CRC (Svrcek et al., 2018). We speculate that EVO significantly decreased the number and size of AOM/DSS-induced colorectal tumors, promoted cell apoptosis and inhibited the proliferation of intestinal epithelial cell, which was related to the early inhibitory effect of EVO on gut inflammation.

Several studies have suggested that gut microbiota play an important role in maintaining intestinal homeostasis, which is closely associated with the pathogenesis of IBD and CRC (Wong and Yu, 2019; Caruso et al., 2020). For example, there are differences in microbiota composition between the inflamed and non-inflamed colonic segments in CD and UC. The transfer of feces from CRC patients to mice enhanced intestinal cell proliferation in germ-free mice and promoted tumorigenesis in conventional mice given AOM to induce colon neoplasia (Wong et al., 2017; Ryan et al., 2020). In view of these, we hypothesize that gut microbiota play a critical role in EVO’s inhibition of enteritis-intestinal cancer. In the present study, we found that EVO could regulate the composition of gut microbiota in AOM/DSS-induced colitis. At the phylum level, the abundance of Proteobacteria was significantly increased in AOM/DSS group. Proteobacteria is enriched in intestinal pathogens, which can cause inflammation and change intestinal microbiota, and promote the development of IBD (Mukhopadhya et al., 2012). Genus of *Alloprevotella*, *Lachnoclostridium* and *Oscillibacter* levels were obviously increased by EVO treatment. *Alloprevotella* is a SCFAs-producing bacteria that plays a key role in maintaining intestinal homeostasis and is believed to confer benefits upon host well-being (Shang et al., 2018; Liu YJ. et al., 2020). *Lachnoclostridium* is involved in the production of propionate, and *Oscillibacter*, which has previously negatively correlated with CD, expresses acetyl-CoA acetyltransferase and enzymes necessary for butyrate production (Mondot et al., 2011; Polansky et al., 2015; Xiang et al., 2021). In the present study, we found that EVO significantly suppressed the increase in the abundance of *Bacteroides*, *Parasutterella* and *Turicibacter* in AOM/DSS-treated colitis mice. It has been reported that the concentration of *Bacteroides* in UC is significantly higher than that in healthy controls, which plays an important role in triggering colitis (Ohkusa and Koido, 2015). The pathogenic Gram-negative bacteria *Parasutterella* was found to be higher in IBD patients with CD and CRC patients (Ibrahim et al., 2019; Sobhani et al., 2019). *Turicibacter* is an opportunistic pathogen or pro-inflammatory bacteria, which was observed an increased in the abundance in AOM/DSS-induced CRC, while some reported that *Turicibacter* was considered an anti-inflammatory taxon as studies proved as depletion of *Turicibacter* in animal models of IBD (Wu et al., 2019; Liu M. et al., 2020). Thus, the role of *Turicibacter* in the development of CRC needs to be further investigated. Moreover, correlation analysis revealed that *Bacteroides* and *Parasutterella* were positively correlated with inflammatory factors and negatively correlated with intestinal barrier integrity, while *Alloprevotella* was the opposite. Microbial dysbiosis and intestinal barrier dysfunction are considered to be two important pathogenic factors that trigger colitis (Jin et al., 2021). Our study showed that EVO inhibited the AOM/DSS-induced colitis through regulating the gut microbiota.

Considering the important role of gut microbiota in CAC development, we further analyzed the changes of gut microbiota in AOM/DSS-induced colon tumor. Notably, EVO could increase the microbial community diversity and change the composition of microbiota. *Prevotellaceae* (the family and the genus *Prevotellaceae_UCG-001*), were induced to decrease by AOM/DSS, which is consistent with our results (Wu et al., 2019). *Prevotellaceae* is identified as a probiotic that is highly coated by secretory IgA, and butyrate could protect the host against colitis-associated colon cancer by enriching its abundance (Zhang Y. et al., 2018). Sutterellaceae*,* which is very commom and abundant in intestinal mucosa, was significantly increased by EVO treatment, and its role largely unexplored with being partly controversial for published reports. Some studies have reported that Sutterellaceae has a mild pro-inflammatory capacity in gastrointestinal tract but not contributing to be disrupted epithelial homeostasis, and the ability of Sutterellaceae to adhere to epithelial mucosa indicate that they may have an immunonodulatory role (Hiippala et al., 2016). The role of Sutterellaceae in the development of CAC deserves further study. *Alistipes* was found to be associated with CRC in a previous study and exhibited increased tumor development (Dai et al., 2018). In the comparison of key bacteria changes between enteritis-intestinal cancer mice treated with EVO, *Ruminococcaceae*, *Alloprevotella*, *Oscillibacter* and *Lachoclostridium* were four beneficial bacteria with the most significant increase from AOM/DSS-induced colitis mice treated EVO, and Sutterellaceae, *unclassified_f_Prevotellaceae*, *Parasutterella* and *Anaeroplasma* were four beneficial bacteria with the most significant increase from AOM/DSS-induced colon tumor mice treated EVO. *Ruminococcaceae*, *Alloprevotella*, *Oscillibacter* and *Lachoclostridium* were considered an anti-inflammatory factor due to their production of SCFAs, which a major source of energy for colonic epithelial cells and exhibits anti-inflammatory properties (Ohkusa and Koido, 2015; Bian et al., 2019). This result indicated that EVO inhibited AOM/DSS-induced colitis by promoting the enrichment of SCFAs producing bacteria to relieve gut inflammation and maintain intestinal barrier. Although some studies have shown an increase in the abundance of *Parasutterella* in CRC, our understanding of the function of *Parasutterella* remains limited. *Parasutterella* isolates successfully colonized the mature mouse gut without causing significant shifts in bacterial composition and host innate immune response, but significantly altered the cecal metabolome, including multiple biological processes and pathways, which may exert potential beneficial effects on the intestinal mucosal homeostasis (Ju et al., 2019). The level of *Anaeroplasma* (phylum Tenericutes) was significantly decreased in obese mice, while the increased abundance is related to a reduction expression of inflammatory factors (Terzo et al., 2020). At the same time, the relative abundance of *Anaeroplasma* was reduced in DSS-induced mice (Zhang XJ. et al., 2018). Sutterellaceae, *unclassified_f_Prevotellaceae*, *Parasutterella* and *Anaeroplasma* forms a beneficial core bacteria set that promotes EVO’s mitigation of AOM/DSS-induced colon cancer. Notable, the relative abundance of *Clostridiales*, *Clostridiaceae* and *Clostridium_sensu_stricto_1* (belong to Firmicutes) were significantly increased from colitis to cancer by EVO treatment, which are associated with inflammation and disease and decreased during inflammation, indicating that it may be the core bacteria of EVO that inhibit CAC development (Zijlmans et al., 2015; Busbee et al., 2020).

The functional capabilities of the microbial communities were analyzed by Tax4fun. We found that EVO treatment may have an impact on a wide range of biological functions. Abnormal lipid metabolism plays a critical role in the progression of adenomas, EVO could significantly decrease lipid accumulation by affecting bile acid biosynthesis and improving bile metabolism to prevent colon cancer (Li et al., 2019). To satisfy the higher requirements of purine and pyrimidine metabolism for tumor growth, nucleotide metabolism is over-transcribed (Chen et al., 2020). *S.cerevisiae* enhances host purine metabolism in colitis mice, and rhein could change purine metabolism to alleviate colitis, which was consistent with our results. Accurate duplication of DNA prior to cell division is essential to suppress mutagenesis and tumor development, while increased DNA replication stress plays a positive role in the proliferation of CRC (Church et al., 2013). It is known that the biosynthesis of lipopolysaccharide, a pathogen-associated pathway, is harmful for the maintenance of the physiological homeostasis, which was inhibited in EVO group (Yao et al., 2017). Additionally, EVO treatment could promote the functional maturation of gut microbiota, which is characterized by increased basic metabolism (carbohydrate metabolism, amino acid metabolism, metabolism of cofactors and vitamins, energy metabolism and cell motility). The metabolism associated with SCFAs (e.g. Butanoate metabolism and Propanoate metabolism) is consistent with the increase of SCFAs-producing bacteria. Reduced levels of amino acid metabolism, such as tryptophan metabolism, are associated with a compromised epithelial barrier in IBD and tumour progression in CRC, indicating that changes in intestinal amino acids metabolism mediated by microbiota may be involved in the pathogenesis of CAC development (Crotti et al., 2019). These data indicate that EVO may mitigate the development of CAC through regulating the function of gut microbiota.

A total of 242 differential metabolites were detected in the three groups through untargeted metabolomics, and these metabolites were involved in KEGG metabolic pathways and mainly belong to the four KEGG modules: metabolism, organismal systems, environmental information processing and human diseases. KEGG pathway enrichment analysis found 12 significant pathways. Biosythesis of other secondary metabolites (Biosythesis of phenylpropanoids and Isoflavonoid biosynthesis) were significantly changed by EVO. Flavonoids are commonly ingested diet-derived compounds that are metabolized by gut microbiota, which have been widely demonstrated to have multiple pharmacological properties in intestinal pathology (Braune and Blaut, 2016; Gong et al., 2019). The microbiome contribution to intestinal flavonoid levels was evident from the elevated levels of naringenin and apigenin in EVO-treated CAC mice. Choline metabolism has been shown to be sensitive to changes in gut microbiota, and is closely connection with colorectal cancer development (Saeedi et al., 2020). Sphingolipids are a class of plasma-associated lipids produced by the host and specific bacteria, which have important biologic functions in metabolic, apoptotic and inflammatory pathways of host cells (Schirmer et al., 2019). Many studies have shown that the abnormalities of sphingolipid metabolism may be involved in inflammation and carcinogenesis, and EVO interferes with changes in sphingolipid metabolism, suggesting that sphingolipid metabolism plays an important role in EVO inhibiting the development of CAC (Ohnishi et al., 2017). EVO treatment also changed the tryptophan metabolism, which is consistent with the prediction of the metabolite function of Tax4Fu, and regulating tryptophan metabolism is also an attractive strategy for the treatment of intestinal diseases.

Based on the results of transcriptomic analysis, we found CAC mice had distinct gene expression profiles compared to Control mice, which may be related to host genetic differences or microbiome differences. Treatment with EVO significantly altered colon gene expression by upregulating and downregulating specific sets of genes. Among the GO terms and KEGG pathways that were significantly enriched by EVO treatment, those involved in tumor-specific immune responses were downregulated. NOD-like receptor proteins have been shown to be associated with various autoimmune or inflammatory diseases, and NOD-like receptor signaling pathways are strongly enriched in colonic tumor tissues (Zhang et al., 2017). Wnt signaling pathway plays a key role in cell development, differentiation and tissue homeostasis, abnormal activation of Wnt signaling pathway is related to the progression of tumorigenesis and tumor metastasis (Zhao et al., 2018). EVO reduced tumor-specific immune responses and may help alleviated CAC. In addition, EVO treatment upregulated pathways involved in signal transduction and Calcium signaling pathway. G protein-coupled receptor ligands, that are encoded by the microbiota and are agonists to receptors (e.g. SCFAs) that have important implications for gastrointestinal and metabolic physiology, has been shown to function as a tumor suppressor in the colon (e.g. GPR109A) (Cani and Jordan, 2018; Lavelle and Sokoi, 2020). In sum, during the procession of intestinal inflammation to carcinogenesis, a variety of immunological and inflammatory signaling events, were inhibited, and a variety of signal transduction and metabolism were activated by EVO and involved in a complex biological process.

In conclusion, we have confirmed that EVO treatment effectively attenuated AOM/DSS-induced colitis and colon cancer in mice by inhibiting the intestinal inflammation and improving mucosal barrier functions. In addition, EVO promoted the enrichment of SCFAs-producing bacteria and reduced the pro-inflammatory bacteria levels, which contributes to the changes of microbiota metabolism, especially tryptophan metabolism. Finally, our results indicated that inflammatory response and tumor-specific immune responses were effectively alleviated by EVO. Collectively, our results highlight that EVO, as a compound with properties in modulating gut microbiota and metabolism, in treating intestinal disorders in the future.

## Data Availability

The original contributions presented in the study are publicly available. This data can be found here: National Center for Biotechnology Information (NCBI) BioProject database under accession number PRJNA770849.

## References

[B1] Allaire J. M. Crowley S. M. Law H. T. Chang S.-Y. Ko H.-J. Vallance B. A. (2018). The Intestinal Epithelium: central Coordinator of Mucosal Immunity. Trends Immunol. 39, 677–696. 10.1016/j.it.2018.04.002 29716793

[B2] Asshauer K. P. Wemheuer B. Daniel R. Meinicke P. (2015). Tax4Fun: Predicting Functional Profiles from Metagenomic 16S rRNA Data. Bioinformatics 31, 2882–2884. 10.1093/bioinformatics/btv287 25957349 PMC4547618

[B3] Bian X. Wu W. Yang L. Lv L. Wang Q. Li Y. (2019). Administration of Akkermansia Muciniphila Ameliorates Dextran Sulfate Sodium-Induced Ulcerative Colitis in Mice. Front. Microbiol. 10, 2259. 10.3389/fmicb.2019.02259 31632373 PMC6779789

[B4] Braune A. Blaut M. (2016). Bacterial Species Involved in the Conversion of Dietary Flavonoids in the Human Gut. Gut. Microbes 7, 216–234. 10.1080/19490976.2016.1158395 26963713 PMC4939924

[B5] Busbee P. B. Menzel L. Alrafas H. R. Dopkins N. Becker W. Miranda K. (2020). Indole-3-carbinol Prevents Colitis and Associated Microbial Dysbiosis in an IL-22-dependent Manner. JCI. Insight. 5, e127551. 10.1172/jci.insight.127551 PMC7030851 31941837

[B6] Cani P. D. Jordan B. F. (2018). Gut Microbiota-Mediated Inflammation in Obesity: a Link with Gastrointestinal Cancer. Nat. Rev. Gastroenterol. Hepatol. 15, 671–682. 10.1038/s41575-018-0025-6 29844585

[B7] Caruso R. Lo B. C. Núñez G. (2020). Host-microbiota Interactions in Inflammatory Bowel Disease. Nat. Rev. Immunol. 20, 411–426. 10.1038/s41577-019-0268-7 32005980

[B8] Chen J. Pitmon E. Wang K. (2017). Microbiome, Inflammation and Colorectal Cancer. Semin. Immunol. 32, 43–53. 10.1016/j.smim.2017.09.006 28982615

[B9] Chen Y. Ni J. Gao Y. Zhang J. Liu X. Chen Y. (2020). Integrated Proteomics and Metabolomics Reveals the Comprehensive Characterization of Antitumor Mechanism Underlying Shikonin on colon Cancer Patient-Derived Xenograft Model. Sci. Rep. 10, 14092. 10.1038/s41598-020-71116-5 32839531 PMC7445290

[B10] Church D. N. Briggs S. E. Palles C. Domingo E. Kearsey S. J. Grimes J. M. (2013). DNA Polymerase ε and δ Exonuclease Domain Mutations in Endometrial Cancer. Hum. Mol. Genet. 22, 2820–2828. 10.1093/hmg/ddt131 23528559 PMC3690967

[B11] Cooper H. S. Murthy S. N. Shah R. S. Sedergran D. J. (1993). Clinicopathologic Study of Dextran Sulfate Sodium Experimental Murine Colitis. Lab. Invest. 69, 238–249. 8350599

[B12] Crotti S. Bedin C. Bertazzo A. Digito M. Zuin M. Urso E. D. (2019). Tryptophan Metabolism as Source of New Prognostic Biomarkers for FAP Patients. Int. J. Tryptophan. Res. 12, 1178646919890293. 10.1177/1178646919890293 31798304 PMC6868567

[B13] Dai Z. Coker O. O. Nakatsu G. Wu W. K. K. Zhao L. Chen Z. (2018). Multi-cohort Analysis of Colorectal Cancer Metagenome Identified Altered Bacteria across Populations and Universal Bacterial Markers. Microbiome 6, 70. 10.1186/s40168-018-0451-2 29642940 PMC5896039

[B14] Desai M. S. Seekatz A. M. Koropatkin N. M. Kamada N. Hickey C. A. Wolter M. (2016). A Dietary Fiber-Deprived Gut Microbiota Degrades the Colonic Mucus Barrier and Enhances Pathogen Susceptibility. Cell 167, 1339–e21. 10.1016/j.cell.2016.10.043 27863247 PMC5131798

[B15] Drewes J. L. Housseau F. Sears C. L. (2016). Sporadic Colorectal Cancer: Microbial Contributors to Disease Prevention, Development and Therapy. Br. J. Cancer 115, 273–280. 10.1038/bjc.2016.189 27380134 PMC4973155

[B16] Gagnière J. Raisch J. Veziant J. Barnich N. Bonnet R. Buc E. (2016). Gut Microbiota Imbalance and Colorectal Cancer. World J. Gastroenterol. 22, 501–518. 10.3748/wjg.v22.i2.501 26811603 PMC4716055

[B17] Gao X. Fan W. Tan L. Shi Y. Ding C. Liu S. (2020). Soy Isoflavones Ameliorate Experimental Colitis by Targeting ERα/NLRP3 Inflammasome Pathways. J. Nutr. Biochem. 83, 108438. 10.1016/j.jnutbio.2020.108438 32563803

[B18] Gong Y. Dong R. Gao X. Li J. Jiang L. Zheng J. (2019). Neohesperidin Prevents Colorectal Tumorigenesis by Altering the Gut Microbiota. Pharmacol. Res. 148, 104460. 10.1016/j.phrs.2019.104460 31560944

[B19] Gong Z. Zhao S. Zhou J. Yan J. Wang L. Du X. (2018). Curcumin Alleviates DSS-Induced Colitis via Inhibiting NLRP3 Inflammsome Activation and IL-1β Production. Mol. Immunol. 104, 11–19. 10.1016/j.molimm.2018.09.004 30396035

[B20] Hiippala K. Kainulainen V. Kalliomäki M. Arkkila P. Satokari R. (2016). Mucosal Prevalence and Interactions with the Epithelium Indicate Commensalism of Sutterella Spp. Front. Microbiol. 7, 1706. 10.3389/fmicb.2016.01706 27833600 PMC5080374

[B21] Ibrahim A. Hugerth L. W. Hases L. Saxena A. Seifert M. Thomas Q. (2019). Colitis-induced Colorectal Cancer and Intestinal Epithelial Estrogen Receptor Beta Impact Gut Microbiota Diversity. Int. J. Cancer 144, 3086–3098. 10.1002/ijc.32037 30515752 PMC6519213

[B22] Jin G. Tang Q. Ma J. Liu X. Zhou B. Sun Y. (2021). Maternal Emulsifier P80 Intake Induces Gut Dysbiosis in Offspring and Increases Their Susceptibility to Colitis in Adulthood. mSystems 6, e01337–20. 10.1128/mSystems.01337-20 33727402 PMC8547008

[B23] Ju T. Kong J. Y. Stothard P. Willing B. P. (2019). Defining the Role of Parasutterella, a Previously Uncharacterized Member of the Core Gut Microbiota. ISME. J. 13, 1520–1534. 10.1038/s41396-019-0364-5 30742017 PMC6776049

[B24] Lavelle A. Sokol H. (2020). Gut Microbiota-Derived Metabolites as Key Actors in Inflammatory Bowel Disease. Nat. Rev. Gastroenterol. Hepatol. 17, 223–237. 10.1038/s41575-019-0258-z 32076145

[B25] Li L. Li X. Zhong W. Yang M. Xu M. Sun Y. (2019). Gut Microbiota from Colorectal Cancer Patients Enhances the Progression of Intestinal Adenoma in Apcmin/+ Mice. EBioMedicine 48, 301–315. 10.1016/j.ebiom.2019.09.021 31594750 PMC6838415

[B26] Li X. Liu Y. Wang Y. Li X. Liu X. Guo M. (2020). Sucralose Promotes Colitis-Associated Colorectal Cancer Risk in a Murine Model along with Changes in Microbiota. Front. Oncol. 10, 710. 10.3389/fonc.2020.00710 32582527 PMC7286428

[B27] Liang X. Li H. Tian G. Li S. (2014). Dynamic Microbe and Molecule Networks in a Mouse Model of Colitis-Associated Colorectal Cancer. Sci. Rep. 4, 4985. 10.1038/srep04985 24828543 PMC4021569

[B28] Liu M. Xie W. Wan X. Deng T. (2020b). Clostridium Butyricum Modulates Gut Microbiota and Reduces Colitis Associated colon Cancer in Mice. Int. Immunopharmacol. 88, 106862. 10.1016/j.intimp.2020.106862 32771947

[B29] Liu Y. J. Tang B. Wang F. C. Tang L. Lei Y. Y. Luo Y. (2020a). Parthenolide Ameliorates colon Inflammation through Regulating Treg/Th17 Balance in a Gut Microbiota-dependent Manner. Theranostics 10, 5225–5241. 10.7150/thno.43716 32373209 PMC7196297

[B30] Mondot S. Kang S. Furet J. P. Aguirre de Carcer D. McSweeney C. Morrison M. (2011). Highlighting New Phylogenetic Specificities of Crohn's Disease Microbiota. Inflamm. Bowel Dis. 17, 185–192. 10.1002/ibd.21436 20722058

[B31] Mukhopadhya I. Hansen R. El-Omar E. M. Hold G. L. (2012). IBD-what Role Do Proteobacteria Play? Nat. Rev. Gastroenterol. Hepatol. 9, 219–230. 10.1038/nrgastro.2012.14 22349170

[B32] Ohkusa T. Koido S. (2015). Intestinal Microbiota and Ulcerative Colitis. J. Infect. Chemother. 21, 761–768. 10.1016/j.jiac.2015.07.010 26346678

[B33] Ohnishi T. Hashizume C. Taniguchi M. Furumoto H. Han J. Gao R. (2017). Sphingomyelin Synthase 2 Deficiency Inhibits the Induction of Murine Colitis-Associated colon Cancer. FASEB. J. 31, 3816–3830. 10.1096/fj.201601225RR 28522594

[B34] Polansky O. Sekelova Z. Faldynova M. Sebkova A. Sisak F. Rychlik I. (2015). Important Metabolic Pathways and Biological Processes Expressed by Chicken Cecal Microbiota. Appl. Environ. Microbiol. 82, 1569–1576. 10.1128/AEM.03473-15 26712550 PMC4771310

[B35] Ranganathan P. Jayakumar C. Manicassamy S. Ramesh G. (2013). CXCR2 Knockout Mice Are Protected against DSS-Colitis-Induced Acute Kidney Injury and Inflammation. Am. J. Physiol. Ren. Physiol 305, F1422–F1427. 10.1152/ajprenal.00319.2013 PMC3840251 23986515

[B36] Rieder F. Fiocchi C. Rogler G. (2017). Mechanisms, Management, and Treatment of Fibrosis in Patients with Inflammatory Bowel Diseases. Gastroenterology 152, 340–e6. 10.1053/j.gastro.2016.09.047 27720839 PMC5209279

[B37] Ryan F. J. Ahern A. M. Fitzgerald R. S. Laserna-Mendieta E. J. Power E. M. Clooney A. G. (2020). Colonic Microbiota Is Associated with Inflammation and Host Epigenomic Alterations in Inflammatory Bowel Disease. Nat. Commun. 11, 1512. 10.1038/s41467-020-15342-5 32251296 PMC7089947

[B38] Saeedi B. J. Liu K. H. Owens J. A. Hunter-Chang S. Camacho M. C. Eboka R. U. (2020). Gut-resident Lactobacilli Activate Hepatic Nrf2 and Protect against Oxidative Liver Injury. Cell. Metab. 31, 956–e5. 10.1016/j.cmet.2020.03.006 32213347 PMC7329068

[B39] Sankarasubramanian J. Ahmad R. Avuthu N. Singh A. B. Guda C. (2020). Gut Microbiota and Metabolic Specificity in Ulcerative Colitis and Crohn's Disease. Front. Med. 7, 606298. 10.3389/fmed.2020.606298 PMC7729129 33330572

[B40] Schirmer M. Garner A. Vlamakis H. Xavier R. J. (2019). Microbial Genes and Pathways in Inflammatory Bowel Disease. Nat. Rev. Microbiol. 17, 497–511. 10.1038/s41579-019-0213-6 31249397 PMC6759048

[B41] Shang Q. Jiang H. Cai C. Hao J. Li G. Yu G. (2018). Gut Microbiota Fermentation of marine Polysaccharides and its Effects on Intestinal Ecology: An Overview. Carbohydr. Polym. 179, 173–185. 10.1016/j.carbpol.2017.09.059 29111040

[B42] Siegel R. L. Miller K. D. Goding Sauer A. Fedewa S. A. Butterly L. F. Anderson J. C. (2020). Colorectal Cancer Statistics, 2020. CA. Cancer J. Clin. 70, 145–164. 10.3322/caac.21601 32133645

[B43] Sobhani I. Bergsten E. Couffin S. Amiot A. Nebbad B. Barau C. (2019). Colorectal Cancer-Associated Microbiota Contributes to Oncogenic Epigenetic Signatures. Proc. Natl. Acad. Sci. U S A. 116, 24285–24295. 10.1073/pnas.1912129116 31712445 PMC6883805

[B44] Sokol H. Seksik P. Cosnes J. (2014). Complications and Surgery in the Inflammatory Bowel Diseases Biological Era. Curr. Opin. Gastroenterol. 30, 378–384. 10.1097/MOG.0000000000000078 24840000

[B45] Svrcek M. Borralho Nunes P. Villanacci V. Beaugerie L. Rogler G. De Hertogh G. (2018). Clinicopathological and Molecular Specificities of Inflammatory Bowel Disease-Related Colorectal Neoplastic Lesions: the Role of Inflammation. J. Crohns. Colitis. 12, 1486–1498. 10.1093/ecco-jcc/jjy132 30202940

[B46] Terzo S. Mulè F. Caldara G. F. Baldassano S. Puleio R. Vitale M. (2020). Pistachio Consumption Alleviates Inflammation and Improves Gut Microbiota Composition in Mice Fed a High-Fat Diet. Int. J. Mol. Sci. 21, 365. 10.3390/ijms21010365 PMC6981517 31935892

[B47] Wang L. Tang L. Feng Y. Zhao S. Han M. Zhang C. (2020a). A Purified Membrane Protein from Akkermansia Muciniphila or the Pasteurised Bacterium Blunts Colitis Associated Tumourigenesis by Modulation of CD8+ T Cells in Mice. Gut 69, 1988–1997. 10.1136/gutjnl-2019-320105 32169907 PMC7569398

[B48] Wang M. X. Lin L. Chen Y. D. Zhong Y. P. Lin Y. X. Li P. (2020b). Evodiamine Has Therapeutic Efficacy in Ulcerative Colitis by Increasing Lactobacillus Acidophilus Levels and Acetate Production. Pharmacol. Res. 159, 104978. 10.1016/j.phrs.2020.104978 32485282

[B49] Wang X. Saud S. M. Zhang X. Li W. Hua B. (2019b). Protective Effect of Shaoyao Decoction against Colorectal Cancer via the Keap1-Nrf2-ARE Signaling Pathway. J. Ethnopharmacol. 241, 111981. 10.1016/j.jep.2020.113532 31146002

[B50] Wang Z. Hua W. Li C. Chang H. Liu R. Ni Y. (2019a). Protective Role of Fecal Microbiota Transplantation on Colitis and Colitis-Associated colon Cancer in Mice Is Associated with Treg Cells. Front. Microbiol. 10, 2498. 10.3389/fmicb.2019.02498 31798539 PMC6861520

[B51] Wong S. H. Yu J. (2019). Gut Microbiota in Colorectal Cancer: Mechanisms of Action and Clinical Applications. Nat. Rev. Gastroenterol. Hepatol. 16, 690–704. 10.1038/s41575-019-0209-8 31554963

[B52] Wong S. H. Zhao L. Zhang X. Nakatsu G. Han J. Xu W. (2017). Gavage of Fecal Samples from Patients with Colorectal Cancer Promotes Intestinal Carcinogenesis in Germ-free and Conventional Mice. Gastroenterology 153, 1621–e6. 10.1053/j.gastro.2017.08.022 28823860

[B53] Wu M. Li J. An Y. Li P. Xiong W. Li J. (2019). Chitooligosaccharides Prevents the Development of Colitis-Associated Colorectal Cancer by Modulating the Intestinal Microbiota and Mycobiota. Front. Microbiol. 10, 2101. 10.3389/fmicb.2019.02101 31620100 PMC6759605

[B54] Xiang S. Ye K. Li M. Ying J. Wang H. Han J. (2021). Xylitol Enhances Synthesis of Propionate in the colon via Cross-Feeding of Gut Microbiota. Microbiome 9, 62. 10.1186/s40168-021-01029-6 33736704 PMC7977168

[B55] Yao X. Zhang C. Xing Y. Xue G. Zhang Q. Pan F. (2017). Remodelling of the Gut Microbiota by Hyperactive NLRP3 Induces Regulatory T Cells to Maintain Homeostasis. Nat. Commun. 8, 1896. 10.1038/s41467-017-01917-2 29196621 PMC5711854

[B56] Yu L. Wang Z. Huang M. Li Y. Zeng K. Lei J. (2016). Evodia Alkaloids Suppress Gluconeogenesis and Lipogenesis by Activating the Constitutive Androstane Receptor. Biochim. Biophys. Acta 1859, 1100–1111. 10.1016/j.bbagrm.2015.10.001 26455953

[B57] Zhang X. Wei L. Wang J. Qin Z. Wang J. Lu Y. (2017). Suppression Colitis and Colitis-Associated colon Cancer by Anti-s100a9 Antibody in Mice. Front. Immunol. 8, 1774. 10.3389/fimmu.2017.01774 29326691 PMC5733461

[B58] Zhang X. J. Yuan Z. W. Qu C. Yu X. T. Huang T. Chen P. V. (2018b). Palmatine Ameliorated Murine Colitis by Suppressing Tryptophan Metabolism and Regulating Gut Microbiota. Pharmacol. Res. 137, 34–46. 10.1016/j.phrs.2018.09.010 30243842

[B59] Zhang Y. Kang C. Wang X. L. Zhou M. Chen M. T. Zhu X. H. (2018a). Dietary Factors Modulate Colonic Tumorigenesis through the Interaction of Gut Microbiota and Host Chloride Channels. Mol. Nutr. Food Res. 62. 10.1002/mnfr.201700554 29331105

[B60] Zhao L. C. Li J. Liao K. Luo N. Shi Q. Q. Feng Z. Q. (2015). Evodiamine Induces Apoptosis and Inhibits Migration of HCT-116 Human Colorectal Cancer Cells. Int. J. Mol. Sci. 16, 27411–27421. 10.3390/ijms161126031 26580615 PMC4661889

[B61] Zhao Q. Bi Y. Zhong J. Ren Z. Liu Y. Jia J. (2018). Pristimerin Suppresses Colorectal Cancer through Inhibiting Inflammatory Responses and Wnt/β-Catenin Signaling. Toxicol. Appl. Pharmacol. 386, 114813. 10.1016/j.taap.2019.114813 31715269

[B62] Zhu L. Q. Zhang L. Zhang J. Chang G. L. Liu G. Yu D. D. (2020). Evodiamine Inhibits High-Fat Diet-Induced Colitis-Associated Cancer in Mice through Regulating the Gut Microbiota. J. Integr. Med. 19, 56–65. 10.1016/j.joim.2020.11.001 33277208

[B63] Zijlmans M. A. Korpela K. Riksen-Walraven J. M. de Vos W. M. de Weerth C. (2015). Maternal Prenatal Stress Is Associated with the Infant Intestinal Microbiota. Psychoneuroendocrinology 53, 233–245. 10.1016/j.psyneuen.2015.01.006 25638481

