# Supplementary material for: Amelioration of AOM/DSS-Induced Murine Colitis-Associated Cancer by Evodiamine Intervention is Primarily Associated with Gut Microbiota-Metabolism-Inflammatory Signaling Axis
Source: Front Pharmacol. 2021 Dec 24;12:797605. doi: 10.3389/fphar.2021.797605 (PMC8740177; doi:10.3389/fphar.2021.797605)

Supplementary Material

**Supplementary Figure 1.** **The effect of EVO on gut microbiota in AOM/DSS-induced colitis mice.** (A) Ace index. (B) PD Whole tree. (C) Simpson index. (D-E) Analysis of similarity. (F) The relative abundance of bacteria on Phylum level. (G) Firmicutes/Bacteroidetes ratio.

**
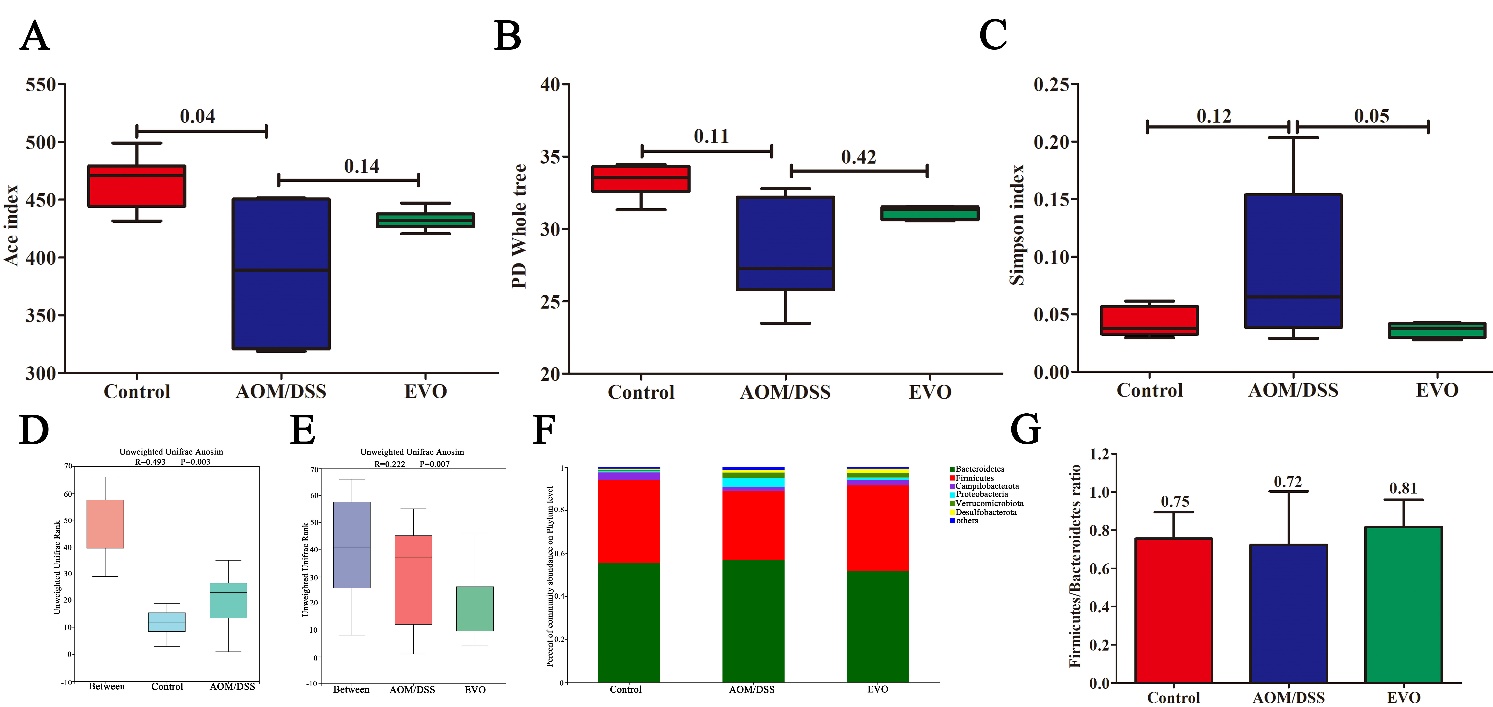
**

**Supplementary Figure 2.** **The effect of EVO on gut microbiota in AOM/DSS-induced colon cancer mice.** (A) Chao index. (B) Ace index. (C) Shannon index. (D) Simpson index. (E) PD Whole tree. (F) Hierarchical clustering tree on OTU level.

**
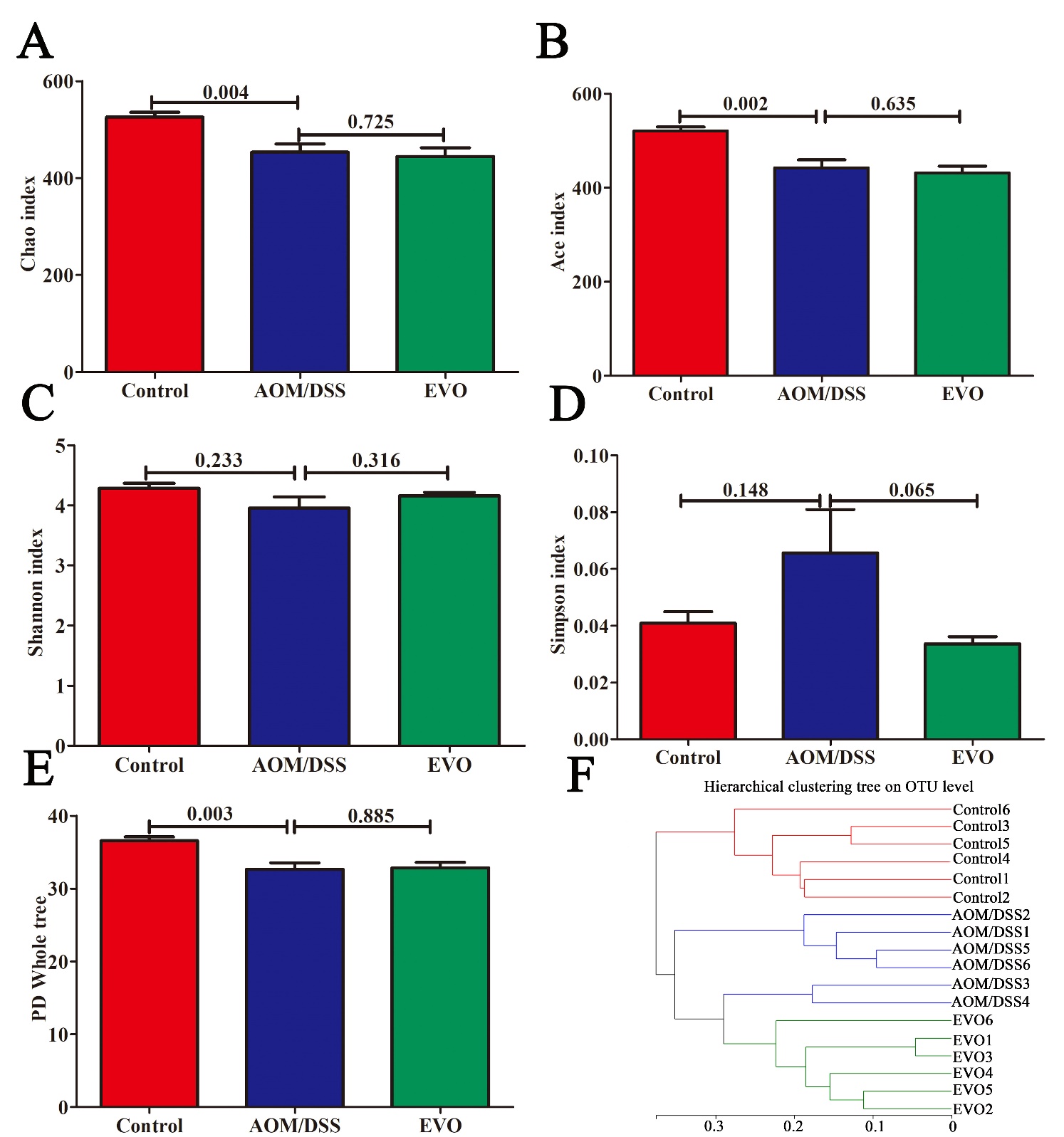
**

**Supplementary Figure 3. LEfSe comparison of microbial communities among Control, AOM/DSS and EVO groups in CAC mice.** LDA scores indicating the size and ranking of the effects for the differentially abundant taxa between Control and AOM/DSS groups, EVO and AOM/DSS groups at 18-days (A-B) and 84-days (C-D).


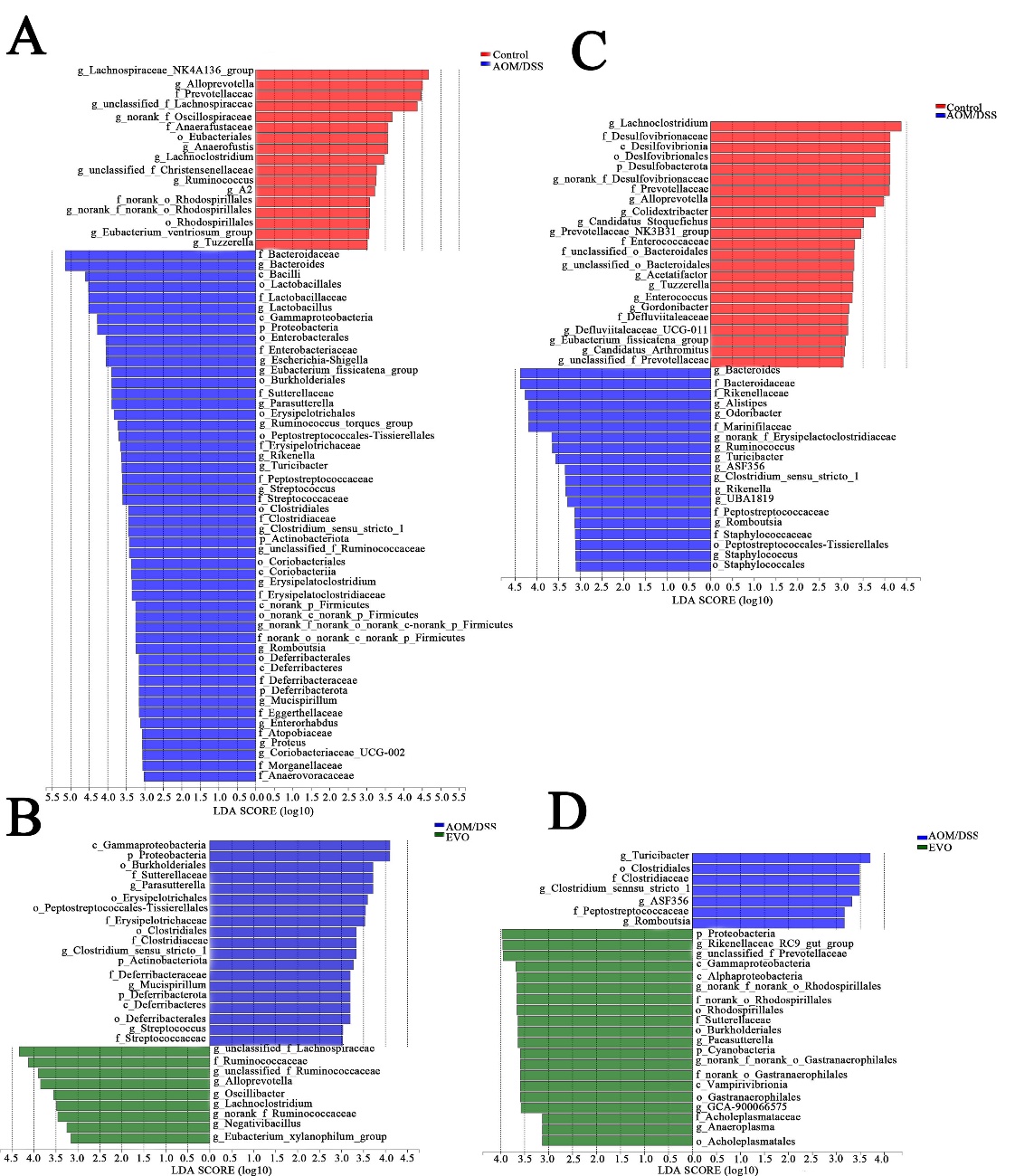


**Supplementary Figure 4.** **LC-MS/MS-based metabonomic analysis of fecal samples.** Heatmap showing the sample similarity on positive (A) and negative (B) mode. PCA score plots on positive (C) and negative (D) mode. Cross validation on positive (E) and negative (F) mode by permutation test.


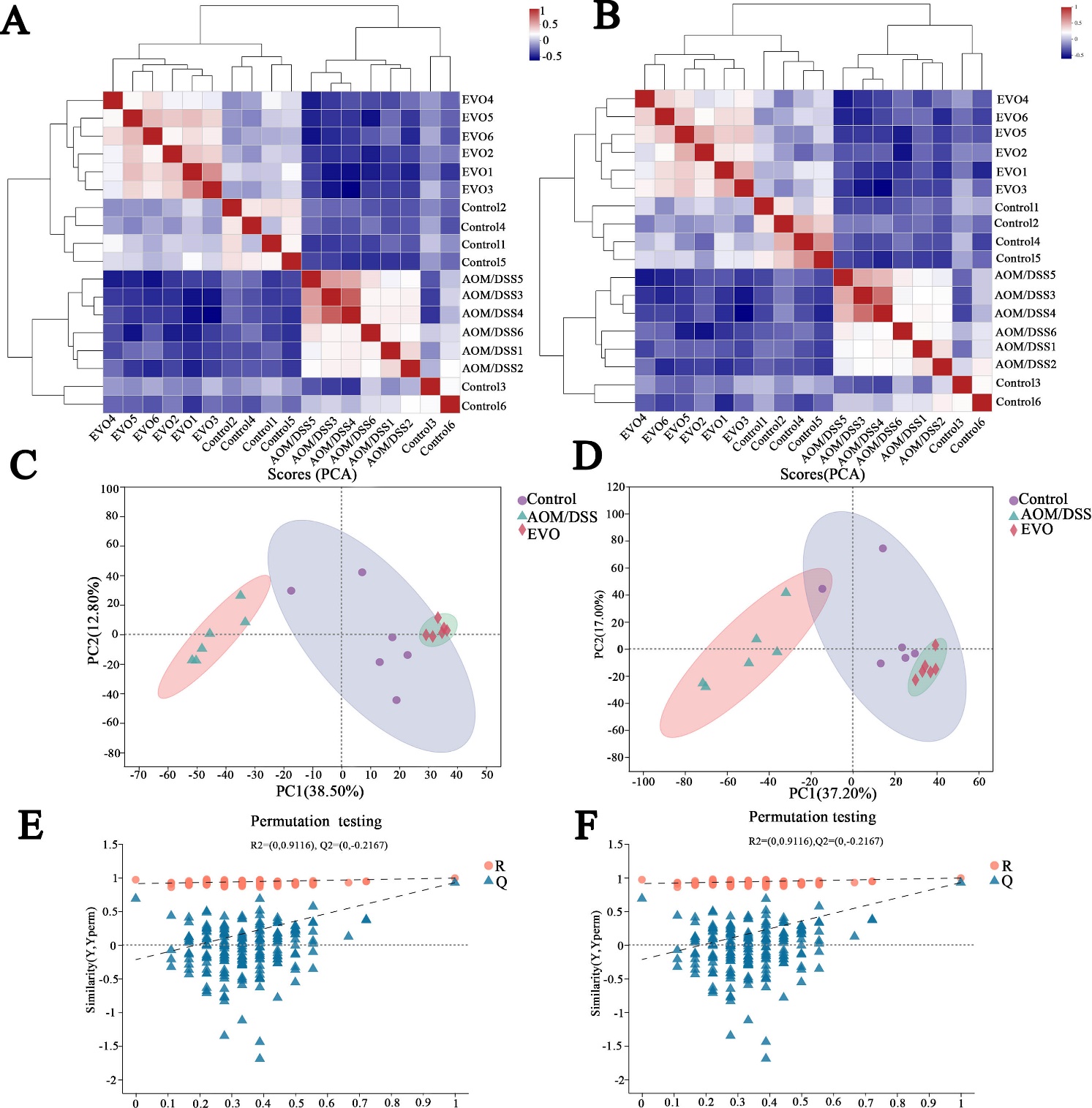

Supplement: Supplementary file 1 [file DataSheet1.docx]
